# Characterisation of an Atrx Conditional Knockout Mouse Model: Atrx Loss Causes Endocrine Dysfunction Rather Than Pancreatic Neuroendocrine Tumour

**DOI:** 10.3390/cancers14163865

**Published:** 2022-08-10

**Authors:** Tiago Bordeira Gaspar, Sofia Macedo, Ana Sá, Mariana Alves Soares, Daniela Ferreira Rodrigues, Mafalda Sousa, Nuno Mendes, Rui Sousa Martins, Luís Cardoso, Inês Borges, Sule Canberk, Fátima Gärtner, Leandro Miranda-Alves, Manuel Sobrinho-Simões, José Manuel Lopes, Paula Soares, João Vinagre

**Affiliations:** 1 Instituto de Investigação e Inovação em Saúde (i3S), University of Porto, 4200-135 Porto, Portugal; 2 Institute of Molecular Pathology and Immunology of the University of Porto (Ipatimup), 4200-135 Porto, Portugal; 3 Institute of Biomedical Sciences Abel Salazar (ICBAS), University of Porto, 4050-313 Porto, Portugal; 4 Faculty of Medicine of the University of Porto (FMUP), 4200-319 Porto, Portugal; 5 Laboratório de Endocrinologia Experimental (LEEx), Instituto de Ciências Biomédicas (ICB), Universidade Federal do Rio de Janeiro, Rio de Janeiro 21941-912, Brazil; 6 Programa de Pós-Graduação em Endocrinologia, Faculdade de Medicina, Universidade Federal do Rio de Janeiro, Rio de Janeiro 21941-905, Brazil; 7 Institute for Molecular and Cell Biology (IBMC), University of Porto, 4200-135 Porto, Portugal; 8 Faculty of Sciences of the University of Porto (FCUP), 4169-007 Porto, Portugal; 9 Department of Endocrinology, Diabetes and Metabolism, Centro Hospitalar e Universitário de Coimbra, 3000-075 Coimbra, Portugal; 10 Centro de Diagnóstico Veterinário (Cedivet), 4200-071 Porto, Portugal; 11 Department of Pathology, Centro Hospitalar Universitário de São João (CHUSJ), 4200-319 Porto, Portugal

**Keywords:** Atrx, conditional mouse model, endocrine fraction, hyperglycaemia, non-alcoholic fatty liver disease, pancreas, pancreatic islet, pancreatic neuroendocrine tumour, Rip-Cre, telomere

## Abstract

**Simple Summary:**

*ATRX* and *DAXX* mutations occur in 30–40% of pancreatic neuroendocrine tumours (PanNETs), and there are no reports in the literature of any genetically engineered mouse model (GEMM) evaluating the effect of *Atrx* disruption as a putative driver event on PanNET initiation. We created a novel GEMM with *Atrx* conditional disruption in β cells. We observed that this genetic alteration, per se, was not tumourigenic, but we reported novel roles of Atrx on endocrine function, which resulted in dysglycaemia and the exacerbation of inflammageing (increased pancreatic inflammation and hepatic steatosis).

**Abstract:**

ATRX is a chromatin remodeller that maintains telomere homeostasis. Loss of *ATRX* is described in approximately 10% of pancreatic neuroendocrine tumours (PanNETs) and associated with poorer prognostic features. Here, we present a genetically engineered mouse model (GEMM) addressing the role of *Atrx* loss (*Atrx^KO^*) in pancreatic β cells, evaluating a large cohort of ageing mice (for up to 24 months (mo.)). *Atrx* loss did not cause PanNET formation but rather resulted in worsening of ageing-related pancreatic inflammation and endocrine dysfunction in the first year of life. Histopathological evaluation highlighted an exacerbated prevalence and intensity of pancreatic inflammation, ageing features, and hepatic steatosis in *Atrx^KO^* mice. Homozygous *floxed* mice presented hyperglycaemia, increased weights, and glucose intolerance after 6 months, but alterations in insulinaemia were not detected. *Floxed* individuals presented an improper growth of their pancreatic endocrine fraction that may explain such an endocrine imbalance. A pilot study of BRACO-19 administration to *Atrx^KO^* mice resulted in telomere instability, reinforcing the involvement of *Atrx* in the maintenance of β cell telomere homeostasis. Thereby, a non-obese dysglycaemic GEMM of disrupted *Atrx* is here presented as potentially useful for metabolic studies and putative candidate for inserting additional tumourigenic genetic events.

## 1. Introduction

The ATRX Chromatin remodeller (*ATRX*) gene was first discovered in patients with α-thalassemia and mental retardation linked to the X chromosome (therefore named ATRX) hereditary syndrome [1]. The prevalence of this condition is extremely low, as only around 200 patients have been identified, and the X-linked recessive genetic inheritance renders it almost exclusive to males [2]. Patients present multiple developmental abnormalities during early childhood, such as severe intellectual disability, sterility, and varying grades of anaemia [3]. *ATRX* is a 300 Kbp-long tumour suppressor that lies at the long arm of X chromosome in position 21.1, composed of 36 exons that encode a 280 kDa full-length protein [4]. ATRX contains two main functional and highly conserved domains: a helicase/adenosine triphosphatase domain, comprising seven helicase motifs and belonging to the SWItch/Sucrose Non-Fermentable (SWI/SNF) family of chromatin remodelling proteins, and a plant homeodomain (PHD)-like zinc finger at the amino-terminus, the ATRX-DNMT3-DNMT3L (ADD) domain [4,5]. ATRX plays multiple functions in the epigenetic landscape [6]. ATRX recruits the death domain-associated protein (DAXX), forming a nuclear chaperone complex that promotes the incorporation of the histone variant H3.3 into telomeric and pericentromeric heterochromatin [7,8,9]. This deposition resolves the accumulation of G- and C-rich repeats that could compromise genomic stability by causing replicative stress and, ultimately, DNA damage [10,11,12].

Within the past decade, as whole-genome and exome sequencing techniques emerged, *ATRX* and *DAXX* mutations were reported in a variety of human tumours, namely various tumours of the central nervous system (CNS) (17% and < 1%, respectively) [13,14,15,16] and pancreatic neuroendocrine tumours (PanNETs) (10% and 20%, respectively) [17,18,19,20]. The prevalence of these mutations stresses their importance as putative tumour driver events.

A correlation between mutations and the telomerase-independent mechanism of alternative lengthening of telomeres (ALT) was established [21,22], with particular importance to PanNETs [23,24,25] that rely on this telomere maintenance mechanism (TMM) two times more often (30%) than the overall tumour types (10–15%, on average) [26]. Losing *ATRX* or *DAXX,* and ALT positivity correlates with chromosome instability, higher tumour grading, and unfavourable prognosis in PanNET patients [23,24,25,27,28].

Genetically engineered mouse models (GEMMs) are still standing out as powerful tools to study the multistep tumourigenic pathway of PanNETs and to evaluate the role of candidate genes on tumour initiation and different stages of tumour progression [29]. Even though the affected main genes and pathways are already identified, there is still limited awareness of their specific role in PanNET tumourigenesis. *MEN1* mutations are the most prevalent alteration in sporadic (35%) [17,18,19] and hereditary (30–80%) [30] human PanNETs. In PanNET GEMMs, *Men1* is well established as a pancreatic endocrine tumourigenesis driver gene. Mutations in other frequent players, such as *Atrx* and *Daxx*, on the other hand, have not been extensively addressed in any context of the PanNET biology. The only report of an *Atrx* knockout (KO), established in a GEMM of pancreatic ductal adenocarcinoma (PDAC), states that Atrx has a potential role in increasing susceptibility to pancreatic inflammation and tumourigenesis [31].

Considering the unpredictability of PanNET behaviour and the never-ending urge to find molecular markers that could anticipate the aggressiveness of these tumours, we developed a conditional mouse model of β-cell specific KO of *Atrx* using the *Rip-Cre* system. We primarily aimed at determining the potential role of *Atrx* as a driver event on PanNET tumourigenesis; however, rather than causing neuroendocrine tumours, we verified that loss of *Atrx* exacerbated ageing-related pancreatic inflammation and hampered endocrine function of KO mice. In this study, we present novel evidence of the role of Atrx in inflammation and pancreatic islets fitness, and its significant impact on weight and glycaemic profiles during the animals’ lifespan.

## 2. Materials and Methods

### 2.1. GEMM Generation

*Atrx^y/f^* male and *Atrx^f/f^* female (*floxed*) mice were gently donated by Professor Douglas R. Higgs and backcrossed with C57BL/6 (B6) by the time of arrival to the animal facility. These mice carry the *Atrx* gene flanked with a *floxed* neo^r^ cassette inserted within intron 17 and *loxP* sites flanking exon 18, as previously reported [32,33,34] (Supplementary File S1: Figure S1). Exon 18 encodes the first of seven motifs that compose the helicase domain of *Atrx.* Its target deletion using the *Cre-loxP* recombination system is demonstrated to severely impair SWI/SNF-dependent gene expression and to highly destabilise the full-length transcript [32,33,35]. This excision still allows for the production of the truncated isoform of Atrx (Atrxt) that arises from transcripts in which there is a failure in splicing intron 11. *Tg(Ins2-cre)25Mgn* (*Rip-Cre*) mice that were obtained from the Jackson Laboratory [36] were crossed with the *Atrx floxed* mice to generate *Atrx^y/wt^;Rip-Cre^+/-^* and *Atrx^wt/wt^;Rip-Cre^+/-^* male and female controls (from now on called “*Atrx^WT^*”), *Atrx^y/f^;Rip-Cre^+/-^* and *Atrx^f/f^;Rip-Cre^+/-^* male and female homozygous individuals (“*Atrx^HOM^*”), and *Atrx^f/wt^;Rip-Cre^+/-^* heterozygous females (“*Atrx^HET^*”). When analysed together, *Atrx^HOM^* and *Atrx^HET^* are designated “*Atrx^KO^*”. Mice were initially backcrossed for genome stabilisation and the colony was submitted to two rederivations during the project, using embryo transplantation into SPF pseudo-pregnant females to reduce the risk of pathogen-related inflammatory states. Animals were genotyped using specific primer pairs (Supplementary File S1: Table S1) by the time of ear tagging.

Animal experimentation was performed in accordance with the Portuguese National Regulation established by Decreto-Lei n.° 113/2013, transposed from the European Directive 2010/63/EU for the Care and Use of Laboratory Animals. Procedures were authorized by the i3S Animal Welfare and Ethics Review Body and the Portuguese National Authority for Animal Health (DGAV)—project license code 13020/2017-05-08. Mice were bred and maintained at the i3S Animal Facility, under a standard 12 h light/dark cycle, with water and rodent maintenance chow diet (Tecklad Global Diet Rodent 2014S, Envigo), available *ad libitum*. The i3S Animal Facility is accredited by the Association for Assessment and Accreditation of Laboratory Animal Care (AAALAC). The authors involved in executing the procedures (T.B.G., S.M., N.M., and J.V.) are certified in animal experimentation (FELASA C course).

### 2.2. Animal Husbandry and Longitudinal Follow-Up

A large cohort of ageing mice was followed up for 24 months (mo.). Throughout the project’s total duration, animals received different and complementary *in vivo* procedures; all procedures, number of animals, and measurements per sex and genotypes are scrutinised in Supplementary File S1: Table S2, according to ARRIVE guidelines 2.0 [37].

During the first exploratory phase of the project (2014–2018, Series 1), animals were let to age and were euthanised when humane endpoints (HEPs) for euthanasia were reached; adopted HEPs consisted in gradual weight loss during three consecutive weightings, dehydration, lethargy, and reluctance to move when stimulated; and clinical evaluation of mice was performed by animal facility staff and researchers (T.B.G., N.M., and J.V.). No procedure rather than euthanasia was performed on animals that reached a HEP; weightings of mice experiencing weight loss before HEP were excluded from weight analysis. Later (2018–2022, Series 2), an in-depth characterisation was carried out, including monthly longitudinal weightings, glycaemia assessment, intraperitoneal glucose tolerance tests (ipGTTs), and hemograms at predetermined time points. When possible, longitudinal data collection was preferred; for data analysis both longitudinal and unpaired data were used. Euthanasia dates were also arranged to obtain an even representation of all age groups by genotypes for histopathological (HP) evaluation. Eight age groups (3, 6, 9, 12, 15, 18, 21, and 24 mo.) were considered for data analyses; the age formula and age range within each group were calculated from dates of birth (DOB) and dates of procedure (DOP) or death (DOD) (see Supplementary File S1: Figure S2). In all the above-stated analyses, a single animal was our experimental unit. The sample size was calculated in G*Power 3.1.9.2, by the time of planning of Series 2.

### 2.3. Euthanasia and Organ Collection

Euthanasia procedures were planned to obtain organ collection and HP evaluations of all age groups. Animals were euthanised using a carbon dioxide chamber or, preferably, via IP injection of ketamine (150 mg/kg) and medetomidine (2 mg/kg), both followed by death confirmation with cervical displacement. The second method allowed for the exsanguination for blood collection to hemogram analysis and/or serum to the ELISA assay. As previously mentioned, at any time of the project, euthanasia was mandatory whenever HEPs were reached. Standard organ collection included the pancreas, spleen, liver, and lungs. To preserve relation with surrounding tissues and allow for further interspecies comparative analysis [38], the pancreas was collected in the block with duodenum, stomach, spleen, and abdominal fat, in which some mesenteric lymph nodes could be present. A complete macroscopic form was filled up in every animals’ necropsy, and each time that any other organ exhibited some pathological alteration.

### 2.4. Histopathological Evaluation

Tissues were fixed in 4% paraformaldehyde (pH 7.4) for 24 h and then routinely processed in an automatic tissue processor for paraffin embedding. Next, 4 µm tissue sections were obtained and stained with haematoxylin and eosin (H&E).

HP evaluation of the pancreas was performed by human and veterinarian pathologists (T.B.G., I.B., S.C., F.G., and J.M.L.), who discussed and agreed on a final scoring system of inflammatory lesions that includes the following parameters: (1) oedema, (2) fibrosis, (3) loss of lobular pattern, (4) duct/vessel dilation, (5) focal acinar atrophy, (6) peripancreatic chronic inflammation (CI), (7) acinar CI, (8) periductal/perivascular (Pd/Pv) CI, and (9) intra-/peri-islet CI. These parameters were evaluated in a four-level scoring system, from 0 (< 5% altered), to 1 (low-grade lesion, 5–33% altered), 2 (moderate-grade lesion, 33–66% altered), or 3 (high-grade lesion, > 66% altered). The sum of the nine parameters obtained the HP score, and some of the most prevalent parameters were evaluated separately, namely Pd/Pv CI.

Liver tissue was evaluated using the non-alcoholic fatty liver disease (NAFLD) activity score (NAS) as previously reported [39,40]. The following parameters were considered: (1) microvesicular steatosis, (2) macrovesicular steatosis, (3) hypertrophy, (4) lobular inflammatory foci (all lymphocytic foci except at portal location), (5) portal inflammation, (6) microgranulomas, and (7) oedema. All parameters were also evaluated with a four-level scoring system; except for microgranulomas that had their grading system, according to their number of foci per ten 200×-magnification fields (0 corresponding to 0–1 focus, 1 to 2–3 foci, 2 to 4–5 foci, and 3 to > 5 foci), and all the remaining were classified from absent (<5%) to high-grade lesion (>66%), as performed in the pancreata slides. The sum of the seven parameters obtained the NAS.

Tumour slides were also evaluated, and a panel of pan-cytokeratins, vimentin, and CD45 antibodies was used to help determine the most probable phenotype of more undifferentiating lesions. Mum1, CD31, and CD44 antibodies were also used in some cases.

### 2.5. Immunohistochemistry Assays

In all procedures, formalin-fixed, paraffin-embedded (FFPE) 4-μm thickened cuts were deparaffinised and rehydrated according to standard protocols. For most protocols, heat-induced antigen retrieval was performed in a steamer for 40 min, followed by 20 min cooling at room temperature (RT), using either 1× citrate buffer (pH 6.0) (Citrate Buffer 10×, AP-9003-500, Thermo Fisher Scientific, Waltham, MA, USA) or 1× EDTA buffer (pH 10.0) (Epitope Retrieval Solution 10×, RE7119, Novocastra, Sheffield, UK). A list of antibodies and specifications of the respective IHC assays are available in Supplementary File S1: Table S3. Endogenous peroxidase activity was blocked with a 3% hydrogen peroxide, either diluted in PBS 1x (vimentin, pan-cytokeratins, and CD45) for 15 min or in methanol (all the remaining) for 10 min. Before adding the primary antibodies, slides were blocked with normal serum (1:5 in antibody diluent) for 30 min; then, sections were incubated with the appropriate biotinylated secondary antibodies for 30 min followed by avidin/biotin complex formation (Vectastain ABC kit, Vector Laboratories, Burlingame, CA, USA) for 30 min, according to the manufacturer’s instructions. Slides were stained with 3,3′-diaminobenzidine (DAB) chromogenic substrate, and modified Mayer’s haematoxylin was used for counterstaining. After dehydration and clarifying, slides were permanently mounted with a xylene-based mounting medium.

### 2.6. Blood Collection and Hemogram Analyses

Longitudinal blood collections for hemogram analyses were performed in mice under general anaesthesia with isoflurane; a puncture was performed with a 25 G needle in the mandibular vein, and the blood drop was collected to a capillary tube; the total amount of blood (~60 μL) was then dropped inside an EDTA tube and then temporarily stored at 4 °C; within a few hours the blood was gently transferred to an Eppendorf to be read in the ProCyte Dx Haematology Analyser (IDEXX, Westbrook, ME, USA). Hemogram analyses were planned for all genotype groups by 3, 6, 12, and 18 mo. In terminal blood collections, blood was collected to dry tubes, centrifuged at 2000× *g*-force for 10 min, and serum separated and stored at −80 °C. Exclusion criteria of hemogram data for analysis were all situations that could compromise leukocyte differential count as abnormal dot plots (see Supplementary File S1: Figure S3), presence of platelet aggregates, and platelets above 850 K/μL.

### 2.7. Glycaemia Assessment and Glucose Tolerance Tests

All procedures of glycaemia assessment were performed by the same operator (T.B.G.) by the current guidelines [41]. Unpaired and longitudinal single glycaemia measurements were performed in 7 h fasted mice (8 a.m.–3 p.m.) of all age groups. After vasodilation under the UV light for 1 min, mice were positioned in a restrainer, and a puncture with a 25 G needle was performed in a lateral caudal vein; glycaemia was measured with a glucometer Aviva (Accu-Check, Corydon, IN, USA). ipGTTs were performed in 6 h fasted animals (8 a.m.–2 p.m.) by the same operator. A 20% glucose solution was administered at the dose of 1.5 mg/kg. The glycaemic values were measured at times 0 (before) and 15, 30, 60, 90, and 120 min after administration, using the method above and the glucometer. This procedure was performed in all genotype groups at 3, 6, and 12 mo. To reduce stress during the process, the operator handled every animal submitted to ipGTT at least once on the days before. Glycaemia values were considered *prediabetic* when above 150 mg/dL and *diabetic* when above 240 mg/dL.

### 2.8. Endocrine Fraction Evaluation

H&E-stained slides were scanned with NanoZoomer 2.0HT (Hamamatsu, Hamamatsu, Japan) slide scanner at 40× magnification with a resolution of 226 nm/pixel. A deep learning algorithm was trained in HALO^®^ Image Analysis Platform version 3.1 (Indica Labs Inc., Albuquerque, NM, USA) to segment pancreatic islets and the exocrine portion automatically. The segmentation output was post-processed in Fiji [42] and a set of measurements was extracted for the morphological characterisation of pancreatic islets. Pancreatic endocrine fraction (EF) was expressed as EF = EndA/(EndA + ExoA) × 100%, where EndA represents the total endocrine area and ExoA represents the exocrine counterpart. Male and female mice were analysed together, and the results were organised by five age groups (3, 6, 12, 18, and 24 mo.).

### 2.9. ELISA Immunoassay

Quantifying serum insulin was performed using the Mouse Insulin ELISA Kit (RAB0817, Millipore, Burlington, MA, USA), specific for mouse insulin, following the manufacturer’s recommendations. Each serum sample was analysed in duplicates.

### 2.10. BRACO-19 Trial

A subcohort of 2-month-old *Atrx^HET^* and *Atrx^HOM^* mice was administered either BRACO-19 (SML0560, Sigma-Aldrich) (2 mg/kg, SID, Monday to Friday, diluted in distilled water (dH_2_O)) or vehicle (dH_2_O) via IP, for 20 or 40 days, as previously performed [43]. The detailed composition of each treatment group can be consulted in Supplementary File S1: Table S4. Besides weight analysis, the experimental unit of this experience outcome was not the individual mice but the acquired images of pancreatic islets.

### 2.11. Telomere Fluorescence In Situ Hybridisation

For telomere fluorescence in situ hybridisation (Tel-FISH), 4 μm FFPE slides were deparaffinised and rehydrated according to standard protocols. An additional wash step for nuclear permeabilization was performed (1% Tween 20 (T20) for 1 min) before antigen retrieval in the steamer using 1x citrate buffer (pH 6.0) (Citrate Buffer 10×, AP-9003-500, Thermo Fisher Scientific) for 40 min; after these steps, slides were dehydrated in a graded series of ethanol for analysis (75%, 95%, and 100%) and then let to dry. Then, slides were applied with 25 µL of a freshly prepared solution containing Na_2_HPO_4_ (10 mM/µL), NaCl (10 mM/µL), Tris-Base (pH 7.5) (10 mM/µL), deionised formamide (OmniPur, Calbiochem, San Diego, CA, USA) (70%), and the TelC-FITC probe (F1009, Panagene, Daejeon, Korea) (0.8 µg/mL), and denaturated at 80 °C for 5 min in the Dako Hybridizer (Dako Colorado Inc., CO, USA). After a 2 h incubation in a dark humidified chamber, slides were washed with a PNA wash buffer made of formamide (70%) and Tris-Base (pH 7.5) (10 mM/mL) and then incubated with freshly prepared DAPI (1:1000, in PBS) for 20 min. In-between washes were made with PBST (0.1% T20) (pH 7.2–7.4). Slides were dehydrated in graded ethanol series and mounted with VECTASHIELD Hardset Antifade Mounting Medium (Without DAPI) (Vector Laboratories), using 0.13–0.17 mm coverslips. Alcohol and PNA wash buffer dilutions were prepared in DEPC-treated water. This procedure was performed on eight animals.

### 2.12. Telomere Status Assessment

#### 2.12.1. Image Acquisition

Pancreatic islets from Tel-FISH-stained slides of treated and control mice were imaged at a motorised Leica DMI6000 (Leica Microsystems, Wetzlar, Germany) widefield microscope, equipped with a Hamamatsu Orca-FLASH 4.0 sCMOS camera (C11440, Hamamatsu), an HCX PL APO 63x/1.30 GLYC Corr CS objective, and a Leica EL6000 light source (metal-halide lamp), and controlled through the LAS X software (version 3.5.5.19976). Samples were imaged using a 2 × 2 binning. For DAPI and FITC acquisition, the following filter cube sets were used, respectively: AT-Excitation: 340–380; BS: 400; Emission: LP 425; and L5-Excitation: 460/40; BS: 505; Emission: 527/30. A Z-stack was acquired with a 247 nm step size. To ensure accurate telomere volume measurements, 100 nm 488-Yellow-Green Fluorescent beads (F8803, Invitrogen, Waltham, MA, USA) were imaged.

#### 2.12.2. Image Analysis

After removing hot pixels using the Hot and Cold Pixel Remover, acquired images were deconvolved with Huygens Professional version 21.10 (Scientific Volume Imaging, Hilversum, Netherlands). The deconvolution wizard used a theoretical PSF and the Classic Maximum Likelihood Estimation (CMLE) algorithm, with estimated background values of 711 (ch1) and 679 (ch2), with SNR 60 (ch1 and ch2) for 100 (ch1) and 500 (ch2) iterations, respectively. The iteration mode was set to classic and the quality threshold value to 0.01. Bleaching correction and a chromatic shift correction were also applied.

Deconvolved images were imported to Imaris x64 9.6.1 and analysis was performed manually using the Surface and Spots wizards followed by its import to Cell wizard (Supplementary File S1: Figure S4). The spot detection size of 0.810 µm was estimated after beads imaging; the axial resolution was assumed to be one-third of the theoretical axial resolution (0.270 µm), as determined by Abbe’s formulas, and previously used [44]. The number of telomeres per nuclei, telomere volume, and intensity sum were analysed.

### 2.13. Statistical Analysis

Statistical analyses were carried out on Prism 9 for macOS (Version 9.1.1) or IBM SPSS Statistics (Version 28.0). Differences between groups were considered significant at a *p*-value < 0.05. The outliers’ ROUT (1%) method was applied in weight and glycaemia analyses. In all graphs *, **, ***, and **** correspond to *p*-values < 0.05, < 0.01, < 0.001, and < 0.0001, respectively. Pearson’s and Kendall’s correlations were used for inter-parameter comparisons when *n* ≥ 15 in both comparison groups or *n* < 15 in at least one comparison group, respectively (Supplementary File S2: Table S1); interpretation of the correlation coefficients was critically made by Schober et al. [45] (0.10–0.39 negligible, 0.40–0.69 moderate, 0.70–0.89 strong, and 0.90–1.00 very strong correlation).

## 3. Results

### 3.1. Study Population

**Figure 1:**
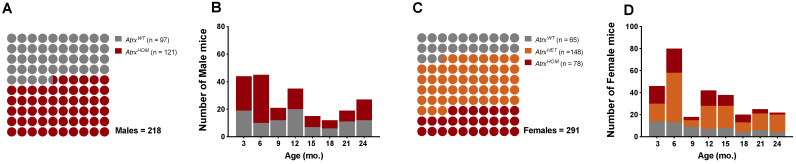
**Study population**. The proportion of genotypes between male (**A**,**B**) and female mice (**C**,**D**), with the respective distribution by the eight age groups. **Grey** means *Atrx^WT^*, **red** means *Atrx^HOM^*, and **orange** means *Atrx^HET^*. Mice ages were calculated by the time of death.

The study population was composed of 509 mice (218 males and 291 females) that were euthanised at different time points, between 2014 and 2022. Mice age by the time of euthanasia (date of death, DOD) was similar between sex groups (mean ± standard deviation (SD)): 11.5 ± 7.3 mo. in males, and 11.3 ± 6.8 mo. in females; age variation was also balanced among male and female genotypes: minimum age varied between 1.9 and 2.5 mo., and maximum ages varied between 27.1 and 27.3 mo. in males and females, respectively. The *Atrx^HOM^* genotype was represented in a proportion of 1:1 to *Atrx^WT^* controls in both sexes; the female population was enriched in the *Atrx^HET^* genotype to allow for more robust comparisons with *Atrx^HOM^* individuals (Figure 1).

### 3.2. Atrx Disruption at β Cells Triggered Anticipation of Local Inflammageing Lesions and Did Not Cause Pancreatic Neuroendocrine Tumours

**Figure 2:**
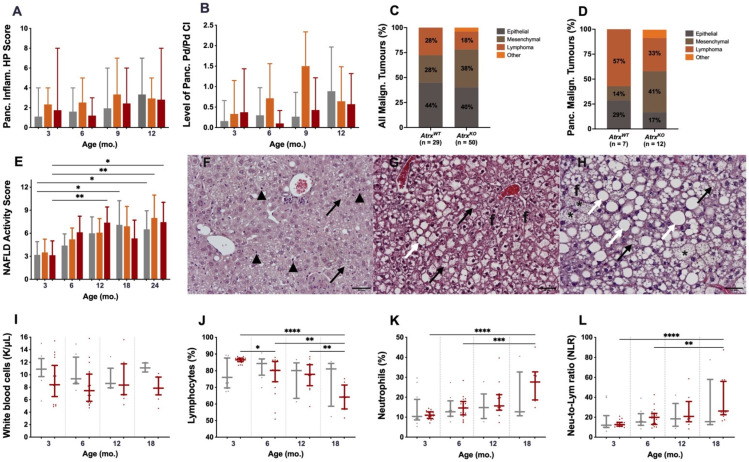
**Inflammatory and tumoural lesions in pancreas, hepatic steatosis, and systemic inflammation in whole blood.** Pancreatic inflammation is higher in *Atrx^KO^* mice, especially *Atrx^HET^* females (**A**), mostly due to increased periductal/perivascular (Pd/Pv) chronic inflammation (CI) (**B**) up to 12 mo.; there are no differences among genotypes. Tumour incidence analysis determined reveal diverse malignant tumoural phenotypes, either considering all tumour sites (**C**) or pancreas alone (**D**), where there is a slight predominance of mesenchymal phenotype in *Atrx^KO^* mice. *Atrx^KO^* mice, especially *Atrx^HET^* females, have increased NAS (**E**), in accordance with weight analysis (See Figure 4). Representative images of mild, moderate, and marked lesions of non-alcoholic fatty liver disease (NAFLD) activity score (NAS) (**F**–**H**), respectively, where **black arrows** represent microvesicular steatosis, **white arrows** represent macrovesicular steatosis, **black arrowheads** represent discrete lobular inflammation, **circles** represent microgranuloma, **asterisks** represent hypertrophy, and **f** represents fibrosis; the three images are at 200× magnification. Hemograms made clearly to conclude inflammation is rather a local phenomenon in pancreas, as all evaluated parameters are not consistently increased in *Atrx^KO^* mice; even so, although white blood cell count is not consistently higher in *Atrx^KO^* mice (**I**), lymphocytes percentage reaches its highest value by 3 mo. and then decreases over time (**J**), accompanied by the inverse pattern in neutrophils population (**K**); from 6 mo. on, the neutrophil-to-lymphocyte ratio (NLR) is slightly higher in *Atrx^KO^* mice than in *Atrx^WT^* controls (**L**). Results are shown as mean +/− range values (**A**), mean +/− SD (**B,E**), and median +/− IQR (**I**–**L**). * *p* < 0.05, ** *p* < 0.01, *** *p* < 0.001, **** *p* < 0.0001.

**Figure 3:**
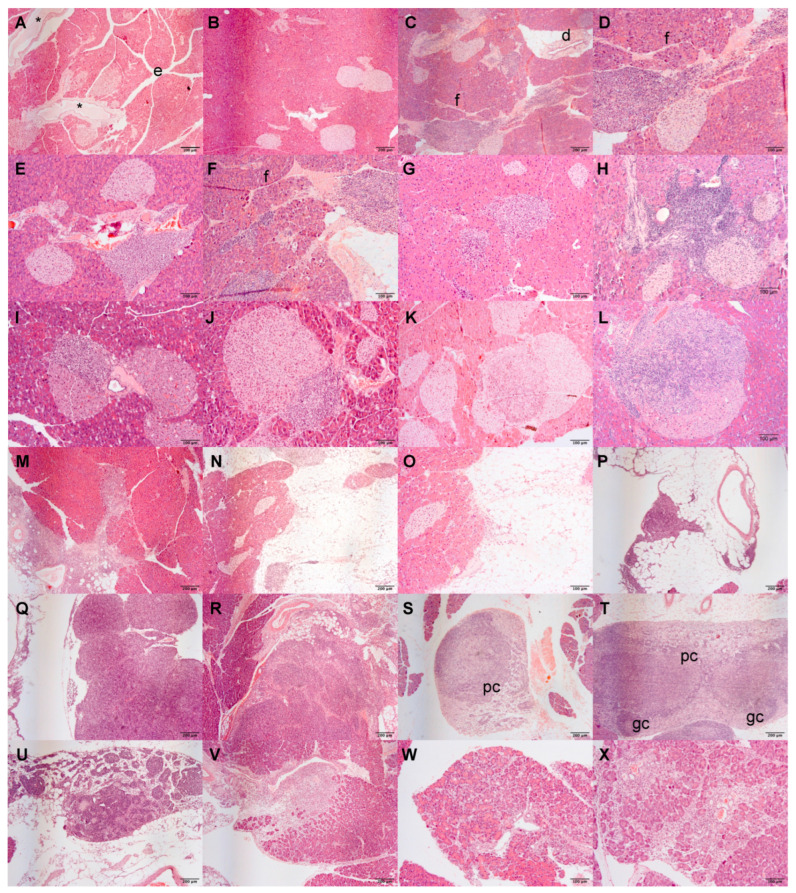
**Pancreatic *inflammageing* lesions.** Histopathological evaluation of our aged cohort reveals a prominent role of inflammation in pancreata of *Atrx^WT^* and *Atrx^KO^* mice. Interlobular oedema (**e**) (**A**), ductal dilation (*), loss of lobular pattern (**B**), ductal dysplasia (**d**) and perivascular inflammation (**C**,**D**), which by continuity is also peri-islet inflammation; also some fibrosis (**f**), perivascular inflammation (**E**), acinar inflammation (**F**–**H**), intra-islet inflammation and islet hyperplasia (**I**–**L**), peripancreatic (pp) inflammation (**M**–**O**), lymphocytes at pp fat invade pancreatic tissue, larger accumulation of lymphocytes in pp fat (**P)**, reactive lymph nodes (**Q**,**R**), paracortical depletion (**pc**) and reduced germinative centres (**gc**) (**S**–**U**), focal acinar atrophy (**W**,**X**). Images (**A**–**C**,**N**,**V**,**W**) are at 40× magnification; remaining images are at 100×.

The pancreata of 289 mice were evaluated with a histopathological (HP) scoring system to characterise chronic inflammation (CI) lesions. Up to 9 mo., *Atrx^KO^* mice presented an increased HP score, especially *Atrx^HET^* females, who exhibited increased periductal/perivascular (Pd/Pv) CI (Figure 2A,B). From 12 mo., all genotypes, including *Atrx^WT^* individuals, presented multiple ageing-related inflammatory lesions that we considered *inflammageing lesions* that hampered discrimination of the lesions cause (age vs. genotype-related) (Supplementary File S2: Figure S1); representative images of such lesions can be consulted in Figure 3. When three age groups (3 mo., 6–12 mo., and 15–24 mo.) stratified the presence of lesions within two genotypes, it was noticeable that, in comparison to age-matched controls, 15–24-month-old *Atrx^KO^* mice presented an increased ageing score, and that 3–12 month-old *Atrx^KO^* increased prevalence and severity of islet CI lesions (Supplementary File S2: Figure S2). We questioned if increased weight profiles could contribute to increased pancreatic CI lesions; we found a strong positive correlation between *Atrx^HOM^* weights and pancreatic HP score. The same tendency is also observed in almost all age groups (Supplementary File S2: Table S1).

A tumour incidence analysis was also carried out. Pancreatic tumours (n = 19) were harmful to neuroendocrine markers, chromogranin A, and synaptophysin, excluding the role of *Atrx* single KO in PanNET development. In total, malignant tumours (n = 79), composed either of primary or secondary tumours, were then evaluated to determine the most likely phenotype. Epithelial tumours were the most common phenotype found in both *Atrx^WT^* and *Atrx^KO^* genotypes, whereas in *Atrx^KO^* mice, most pancreatic tumours were mesenchymal (41%), followed by lymphomas (33%); in *Atrx^WT^* mice, most pancreatic tumours were lymphomas (57%), followed by epithelial tumours (29%) (Figure 2C,D). Tumour incidence was equal between genotypes in male mice (45%) but higher in female mice of *Atrx^KO^* vs. *Atrx^WT^* genotype (40% vs. 29%, respectively) (Supplementary File S2: Table S2).

As an essential feature of ageing mice, hepatic adiposity, was also evaluated in our model, using the non-alcoholic fatty liver disease (NAFLD) activity score (NAS). *Atrx^KO^* mice presented slightly higher NAS than age-matched controls; although no statistically significant differences were found, such a tendency is noted from 6 mo. on, as the *Atrx^HET^* females exhibit the highest scores (Figure 2E). Representative images of mild, moderate, and marked lesions are depicted in Figure 2F–H. More detailed information concerning the evolution of NAS over time and its correlations with weight gains can be consulted in Supplementary File S2: Table S1. By the time of necropsy, macroscopic evaluation evidenced a slight increase in the prevalence of hepatomegaly and splenomegaly (15% and 13% increase, respectively) in *Atrx^KO^* mice of 15 mo. and older than in age-matched *Atrx^WT^* mice (data not shown).

We decided to evaluate if the prominent pancreatic inflammatory lesions would be detected systemically in blood, via longitudinal hemogram analysis. Firstly, total white blood cell (WBC) count was compared with reference values reported by Charles River Laboratories^TM^ for B6 mice, and *Atrx^KO^* individuals did not present any elevation of such a parameter by any time point (data not shown); actually, *Atrx^WT^* mice exhibit slightly higher levels than age-matched *Atrx^KO^* mice (Figure 2I; Supplementary File S2: Table S1). Then, the differential leukocyte count and percentage were evaluated to check for different profiles between genotypes; a complete report of the haematologic parameters of our GEMM can be consulted in Supplementary File S2: Table S3. By 3 mo., the lymphocyte percentage was tendentially increased in *Atrx^HOM^* individuals compared to age-matched controls (86% vs. 76%, median values) (Figure 2J; Supplementary File S2: Table S1). The neutrophil-to-lymphocyte ratio (NLR), assessed due to its potential prognostic relevance in anticipating malignancy, also does not significantly differ between genotypes (Figure 2K,L).

### 3.3. Atrx^KO^ Mice Exhibit Increased Weight Gains and Glycaemia Levels since 6 mo.

**Figure 4:**
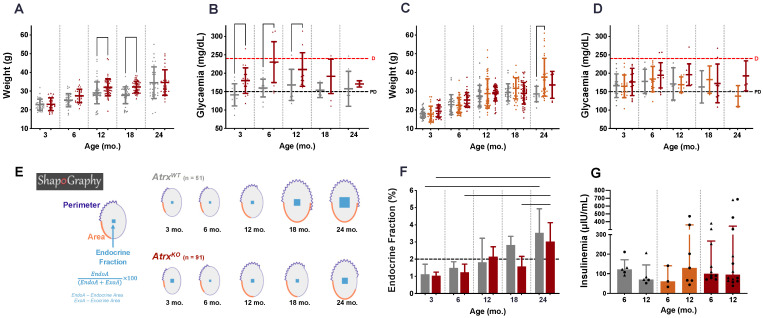
**Endocrine fitness: weight, glycaemia, endocrine fraction, and insulinaemia.** Weight and glycaemia analyses indicate that *Atrx^KO^* male (**A**,**B**) and female (**C**,**D**) mice concurrently present increased weight gains and glycaemia levels than age- and sex-matched controls. Age groups of 9, 15, and 21 mo. were included in 12, 18, and 24 mo. age groups, respectively (**A**–**D**). *Atrx^KO^* mice (**E**,**F**); other morphological parameters like perimeter and area also increase less with ageing (**E**); 6 h fasted insulin levels do not significantly differ among groups (**G**). Results are shown as mean ± SD (**A**–**D**) or median ± IQR (**F**,**G**); triangles and circles are used to represent values of male and female mice, respectively (**G**). **Grey** means *Atrx^WT^*, **red** means *Atrx^HOM^* (**A**–**D**,**G**) or *Atrx^KO^* (**E**,**F**), and **orange** means *Atrx^HET^*.

Considering the inability of *Atrx* single KO at β cells to induce neuroendocrine tumour formation, we decided to search for other possible outcomes, focusing on endocrine metabolism. Therefore, a baseline of weight and glycaemic profiles was determined. A total of 898 weightings (obtained from 347 animals) were considered and distributed along five age groups (3, 6, 12, 18, and 24 mo.); all the available non-longitudinal weights obtained during DOD were also included. Blood glucose levels were obtained at the same time points (3, 6, 12, 18, and 24 mo.), and 241 measurements were used for data analysis. By 3 mo., *Atrx^KO^* mice of both sexes seem to present equivalent weights to age-matched controls; by 6 mo., *Atrx^HOM^* male and female mice tend to show increased weight gain; by 12 mo., and this trend becomes statistically significant in male mice, while the group of *Atrx^HET^* females exhibits the highest average weight (Figure 4A,C). The preponderance of *Atrx^HOM^* genotype to be associated with increased weight gain is remarkably consistent in males, up to 18 mo.; on the other hand, *Atrx^HET^* females are the heaviest individuals in our cohort (Supplementary File S2: Figure S3A,B).

In parallel, *Atrx^KO^* mice of both sexes also present increased glycaemia; such a difference appears earlier in males (3 mo.), with consistent statistical difference up to 12 mo. (Figure 4B), while females start showing a similar tendency months later, from 6 to 18 mo. (Figure 4D); although *Atrx^HET^* females have the highest weights, *Atrx^HOM^* females are the ones with increased glycaemia levels (Supplementary File S2: Figure S3C,D). When data of weight and glycaemia analyses were plotted together in Orange Data Mining [46], we could observe two clusters given by two separate colour regions in male mice, in which higher weights are associated with higher glycaemia. At the same time, in females, such distinction is harder to establish (Supplementary File S2: Figure S3E,F).

A complementary weight and glycaemia parameters analysis is depicted in Supplementary File S2: Table S1. It should be noted that a moderate positive correlation between weights and glycaemia was found for *Atrx^HOM^* genotype both by 3 and by 6 mo. (r = 0.521 and r = 0.423, respectively), and that average weights of *Atrx^HOM^* and *Atrx^HET^* females are statistically different by 6 mo. (higher in *Atrx^HOM^*) and by 9 mo. (higher in *Atrx^HET^*); average glycaemia is also statistically different in female genotypes by 12 mo. (higher in *Atrx^HOM^*). Ageing powerfully contributes to the significant rise of the values of the two parameters, attenuating the differences at older ages.

### 3.4. Atrx^KO^ Mice Show Improper Ageing-Related Growth of the Endocrine Fraction and Similar Fasted Insulinaemia

To complement the weight and glycaemia analysis, a detailed morphological characterisation of pancreatic islets was also performed, as a readout of in situ endocrine fitness, including multiple morphological parameters, namely endocrine fraction (EF), perimeter, and area. Considering the EF as the best functional readout, the *Atrx^KO^* genotype consistently exhibited smaller EF in all age groups, presenting a smaller EF growth (15% less) from 3 to 24 mo. age groups (1.04% to 3.02%, median values) in comparison with *Atrx^WT^* mice (from 1.12% to 3.53%, median values) (Figure 4E,F). Islet number was also not statistically significant between genotypes but was always lower in *Atrx^KO^* mice of all age groups, as the mean islet area follows the same tendency (Supplementary File S2: Figure S4). Despite these results, strong (r = 0.714) and moderate (r = 0.604) positive correlations were found between EF and weight of *Atrx^HOM^* mice by 6 and 12 mo., respectively (Supplementary File S2: Table S1).

At this point, given evidence of endocrine metabolism disturbance of *Atrx^KO^* mice, we questioned if these individuals would present different insulinaemia values than age-matched controls by 6 mo. and 12 mo. Firstly, we used frozen serum collected from non-fasted mice of Series 1 and quantified the insulin levels by ELISA. The results did not indicate any significant difference between genotypes, although a tendency was noted in 6 mo. *Atrx^KO^* male and female mice present increased values than age-matched controls (Supplementary File S2: Figure S5A,B). Then, we included more animals in the cohort to measure insulinaemia after a 6 h fasting period. We observed that, by 12 mo., both *Atrx^HET^* and *Atrx^HOM^* groups exhibited a slight increase in median insulin levels than genotype-matched groups by 6 mo., as some *Atrx^KO^* individuals presented values above 300 μIU/mL. The assay results are presented in Figure 4G (male and female mice analysed together) and in Supplementary File S2: Figure S5C,D (male and female mice analysed separately). Pooling the results of each genotype regardless of mice age, the level of insulin was higher in the *Atrx^HOM^* group (mean ± S.E., 192.5 ± 38.30 μIU/mL, *n* = 24 male and female mice), followed by *Atrx^HET^* females (157.3 ± 46.67 μIU/mL, *n* = 10) and *Atrx^WT^* male and female controls (114.8 ± 17.53 μIU/mL, *n* = 10). The glycaemia/insulinaemia ratios, indicative of insulin resistance (IR), are higher in 12 mo. *Atrx^HOM^* male and female mice than age-matched controls. Of note, the highest median values in females were already detected by 6 mo. in *Atrx^HET^* and *Atrx^HOM^* genotypes (Supplementary File S2: Figure S5E,F).

### 3.5. Atrx^HOM^ Mice Exhibit Frank Glucose Intolerance Already by 6 mo.

**Figure 5:**
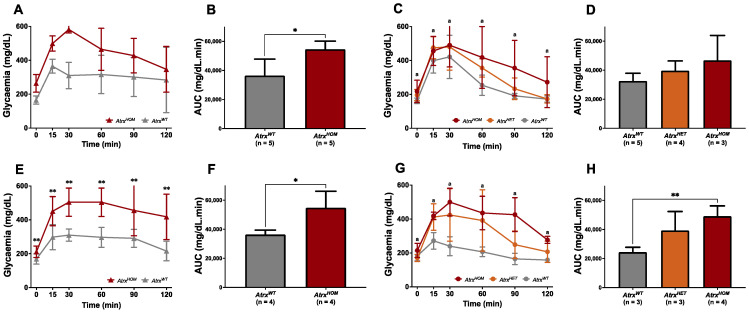
**Evidence of glucose intolerance.** Intraperitoneal glucose tolerance tests (ipGTTs) indicate that, by 6 mo., *Atrx^HOM^* have significantly increased glucose intolerance (**A**,**B**), while females show a similar tendency (**C**,**D**); by 12 mo., both male (**E**,**F**) and female (**G**,**H**) *Atrx^HOM^* mice exhibit significant frank deterioration of glucose tolerance; *Atrx^HET^* females, who are the heaviest, are not the ones with higher glycaemic values (see Figure 4C,D), responding better to ipGTT than *Atrx^HOM^* females; two-way ANOVA used for comparisons in each time point: **a** *p* < 0.01 (*Atrx^HOM^* vs. *Atrx^WT^*) (**C**,**G**). * *p* < 0.05, ** *p* < 0.01.

To confirm the potential role of Atrx loss in endocrine dysfunction, ipGTTs were additionally performed in 3, 6, and 12 mo. male and female mice of all genotypes. It was verified that by 3 mo., mice of all genotypes showed an equivalent response to glucose administration, as they were able to gradually restore normoglycaemia within approximately 90 min; however, *Atrx^HOM^* male mice already started to exhibit a slight glucose intolerance (Supplementary File S2: Figure S6). By 6 mo., *Atrx^HOM^* male mice already exhibited statistically significant glucose intolerance, and by 12 mo., both male and female mice of *Atrx^HOM^* genotype presented frank glucose intolerance. In contrast, *Atrx^HET^* females presented both glucose measurements and AUC between *Atrx^WT^* and *Atrx^HOM^* genotypes (Figure 5A–H).

### 3.6. Atrx^KO^ Individuals Are Sensitive to Exogenous Telomere Provocation

**Figure 6:**
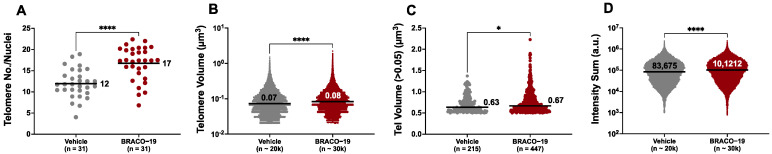
**Evidence of telomere instability upon treatment with BRACO-19. a.u.** signifies arbitrary units. In comparison with *Atrx^KO^* mice administered with vehicle (dH_2_O) (*n* = 4), *Atrx^KO^* mice administered with BRACO-19 (*n* = 4) for 40 days exhibit significantly higher number of telomeres per pancreatic islet cell nuclei (**A**), significantly increased telomere volume (**B,C**), and significantly increased intensity sum of FITC-stained telomeres (**D**). Data of graph A passed the normality tests, an unpaired t-test was then performed, and the mean values are presented. Data of graphs B–D did not pass the normality tests, so Mann–Whitney tests were then performed, and the median values are presented. A Log 10 scale and an Antilog tick format were used for the representation of the intensity sum (**D**). * *p* < 0.05, **** *p* < 0.0001.

Finally, we aimed to provoke telomere interference by administering the G4 stabiliser BRACO-19 to *Atrx^KO^* mice. Telomeres of pancreatic islets nuclei were analysed. We found evidence of telomere instability upon treatment with BRACO-19 for 40 days (Group III, see Supplementary File S1: Table S4). Treated mice present a significantly higher number of telomeres per nuclei, significantly increased telomere volume, and significantly increased telomere intensity sum (20% more) (Figure 6). The nuclei volume of the BRACO-19-treated group was also higher than the vehicle-administered group (10% more) (Supplementary File S2: Figure S7). Normalised data, to the number of analysed images, are also shown in the same figure; although statistical significance is lost, the same tendency of amplified telomere stress/instability is maintained in the group of *Atrx^KO^* mice treated with BRACO-19.

## 4. Discussion

Pancreatic neuroendocrine tumours are rare and clinically challenging entities in which genetic profiling has been progressively unveiled over the last decade. However, there is still limited knowledge of what determines PanNET origin and behaviour. After *MEN1* inherited and acquired mutations, epigenetic changes caused by loss of *ATRX* or *DAXX* were pointed to be as the main driver events in PanNET biology [47]. Losing *ATRX*/*DAXX* is associated with changes in the methylation status of telomeric regions, interspersed repetitive sequences, and several other genome regions, ALT activation, and chromosome instability [47,48,49]. Accumulating evidence in large PanNET cohorts has ascertained the prognostic value of *ATRX*/*DAXX* mutations [23,24,25,27,28].

GEMMs are a versatile tool to study various genetic variations influencing PanNET aetiopathogenesis and progression over time. Conversely, up-to-date GEMMs assessing the loss of function of chromatin remodellers are still the most underrepresented. The potential anti-inflammatory and tumour suppressive role of Atrx has been described in a PDAC GEMM [31]. Still, there are no reports in the literature of the possible involvement of *Atrx* loss as a putative driver event in a PanNET GEMM. Wasylishen *et al.* [50], however, already reported good tolerance for *Atrx* loss in the developing pancreas of mice, using CRISPR-Cas9.

Our primary goal in this work was to assess the potential tumourigenic effect of this genetic alteration. Therefore, after GEMM creation, mice were allowed to age, and we centred our preliminary analysis on HP evaluation. In the beginning, we observed that, regardless of the genotype, mice developed ageing-related pancreatic inflammatory lesions and sporadic non-neuroendocrine (NE) tumours at multiple locations. We enriched the cohort with younger animals to observe genotype-related differences, which were small and apparently only detected at a higher intensity in *Atrx^KO^* individuals, compared to age-matched controls, during the first 12 mo. of the animals’ lifespan, before *inflammageing* establishment in all cohorts. *Inflammageing* has been attributed increasing relevance in ageing studies. It is defined as a subclinical state characterised by chronic, sterile, and low-grade inflammation that plays a role in the development and progression of multiple ageing-related diseases, by contributing to the functional decline of tissues [51,52,53]. In the face of such ageing-related HP alterations and non-NE tumours throughout mice genotypes, we decided to explore collateral non-tumourigenic effects that could be assignable to *Atrx* loss in islet β cells, paying particular attention to the first year of the animals’ lives. Thus, we closely followed mice’s weight and glycaemic profiles longitudinally, including monthly weightings and glycaemia assessment at predetermined time points. Weight analysis revealed that *Atrx^KO^* male and female individuals presented higher weights than age-matched controls after 6 mo., and fasted glycaemia measurements also indicated that *Atrx^KO^* mice consistently exhibited more elevated values, mainly at a prediabetic level (i.e., 150–240 mg/dL), with statistical significance after 3 mo. in *Atrx^HOM^* male mice. This potential endocrine dysfunction led us to perform ipGTTs that confirmed the phenotype of glucose intolerance in our GEMM, which was clear and statistically significant in *Atrx^HOM^* of both sexes by 12 mo. of age.

Mice represent 60% of the used preclinical animal models to investigate metabolic disorders. The inbred C57BL/6J (B6) mouse strain, the background of our mice, is widely used as a model for diet-induced obesity (DIO) due to the tendency to develop severe obesity, elevated adiposity, glucose intolerance, and moderate insulin resistance (IR) [54,55]. We were also aware that sexual dimorphism would likely exist, as male mice are more susceptible to DIO once they develop obesity sooner and to a greater extent than females [55,56]. For this reason, males and females were always analysed separately, and results only joined when no differences were present [57]. As expected, the weighing results in males were more pronounced and detected sooner than in females. Of note, mice of this cohort hardly ever become obese, even in the second year of life. Both *Atrx^WT^* and *Atrx^HOM^* male and female mice present diminished mean weights compared to reference values [58] (data not shown).

The detailed quantitative morphological characterisation of pancreatic islets and the ELISA assays for insulin quantification assist in understanding the aetiopathogenesis of such endocrine dysfunction. Endocrine functions are particularly vulnerable to ageing phenomena. Like in humans, glucose tolerance and insulin sensitivity of mice deteriorate with age, contributing to metabolic disorders [41,57,59]. In response to increased metabolic demand (primarily for insulin), pancreatic islets should undergo a morphological compensation to maintain normoglycaemia, as it is described for both species [60,61,62,63,64]. Such a demand could be anticipated with obesity, in which IR is the primary driver of β-cell mass adaptation [62,65]. We trained a deep-learning algorithm to segment the endocrine and exocrine areas and quantify the pancreatic EF in a subset of our mice. Contrarily to most quantification methodologies, we did not use pre-stained pancreatic islets. Instead, we scanned H&E-stained slides and used the fraction of the islet area in the total area (exocrine tissue and islets) quantified in HALO (Indica Labs) as a readout of endocrine fitness. We noticed that *Atrx^KO^* mice not only did not increase as much as expected their EF in response to higher metabolic demand, but even showed a 15% decreased ability to induce such ageing-related morphological changes in comparison with age- and sex-matched controls, although it was not significant. This suggested a potential increasing inability to deal with the ageing-related rise of insulin demand and could justify the glucose intolerance of *Atrx^KO^* mice.

It is described that old mice still hold capacity for the significant compensatory proliferation of β cells, which results in a duplication of islet size between 3 mo. and 21 mo., and such an increase is enough to sustain normoinsulinaemia and normoglycaemia [63,66]. At the exocrine counterpart, acinar atrophy, fibrosis, and fat accumulation are common ageing-associated changes in mice and men [60,67] that influence EF calculation. Therefore, these results should be interpreted with caution because (a) the quantified endocrine area does not represent solely β cells (~75% of islet cell population in mice), but also represents other endocrine cells, especially peripheric α cells (~18%) [67,68]; (b) a single whole-slide image per mouse was used for morphological quantifications when the most valuable method is stereology, that uses multiple slides per pancreas [69]; and (c) the degree of pancreatic CI and fat accumulation, not accounted for EF calculation, could also be compromising the accuracy of the results as a readout of insulin production fitness. Pancreas plasticity, which renders stem, exocrine, and other endocrine cells the ability to differentiate into β cells [70,71], should also not be excluded from the many possible explanations for the lack of escalation of the endocrine dysfunction.

In addition to EF, the results of insulinaemia were also intriguing. Maybe because animals never got obese by the time of the highest detected glucose intolerance (12 mo.), no statistically significant differences were found on the ELISA assays, regarding either insulinaemia or glycaemia/insulinaemia ratios, indicative of IR. We hypothesised that disruption of Atrx in β cells would somehow interplay with insulin secretion, which would be noticeable upon insulinaemia measurements in fasted mice. Since that did not happen, we believe that the mild metabolic impairment of *Atrx^KO^* mice, particularly evident upon exogenous glucose administration, continued to be easily compensated by functional insulin-producing β cells that maintain the ability of mice to cope with their basal metabolism.

**Figure 7:**
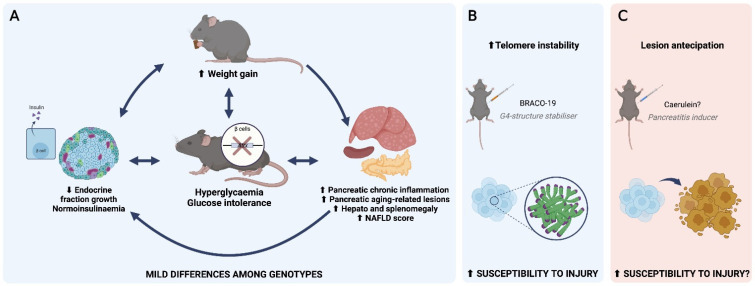
***Atrx^KO^* mice as a non-obese dysglycaemic model.***Atrx* disruption at β cells seems to induce a state of dysglycaemia, characterised by hyperglycaemia and glucose intolerance (**A**); this is accompanied by increased weight gain leading to overweight (not obesity), which could both explain the origin and be a consequence of the impaired metabolism. *Atrx^KO^* mice seem to be unable to double its endocrine fraction with ageing, although insulinaemia values did not seem to be altered. As a consequence of ageing, pancreatic chronic inflammation (CI), ageing-related lesions, and non-alcoholic fatty liver disease (NAFLD) are present in all genotypes with a mild predominance in *Atrx^KO^* individuals. A slight increase of hepato and splenomegaly was noticeable in 15–24-month-old *Atrx^KO^* mice. Genotype-related differences in pancreatic CI scores were only noticeable during the first year of life. Similarly to exogenous glucose administration, BRACO-19 intake also induced changes in *Atrx^KO^* mice, by causing telomere instability, a state that would predispose endocrine pancreas to injury (**B**). We hypothesise if the exogenous administration of a pancreatitis inducer like caerulein would also increase susceptibility to injury allowing for an anticipation of lesion formation in endocrine pancreas, as previously observed (**C**) [50].

We believe that the dysglycaemia presented by *Atrx^KO^* mice, characterised by hyperglycaemia and diminished glucose tolerance, has a multifactorial origin, marked by (1) increased weight gain, (2) improper growth of EF, and (3) development of *inflammageing* lesions (e.g., pancreatic CI infiltrates and ageing-related lesions, and hepatic steatosis, measured by NAS). These three axes will likely interplay in our GEMM, as depicted in Figure 7. Pancreatic inflammation has been linked to obesity, metabolic syndrome, and type 2 diabetes (T2D) [71], and NAFLD alters not only hepatic metabolism, but also muscle, adipose tissue, and the pancreas to induce IR [72]. A higher glycaemia to insulinaemia ratio was verified in 12 mo. *Atrx^HOM^* male mice in comparison to 6 mo. sex- and genotype-matched individuals.

The analysis of the *Atrx^HET^* females is interesting, since animals of this genotype group were the only ones in the cohort developing obesity in the second year of life. This is consistent with the highest NAS, and pancreatic HP inflammatory score detected in this genotype, which may be attributable to the NAFLD-related lipotoxicity and higher adiposity [72,73]. What is curious is that *Atrx^HET^* females were more competent in endocrine function than age-matched *Atrx^HOM^* females. This may indicate that *Atrx* loss in both alleles mainly harms the endocrine competence and only indirectly contributes to the aggravation of physiological ageing-related pancreatic inflammation. It remains to understand why differences in glycaemic profiles are more accentuated than weights, suggesting that hyperglycaemias are not caused by overweight or IR. It also remains to be determined why *Atrx^KO^* mice, who have an impairment of endocrine metabolism, have increased weight gain.

To validate the functional compromise of Atrx caused by our mouse modelling, a subcohort of mice was treated with BRACO-19, a G4-structure stabiliser. We could observe that treated *Atrx^KO^* mice presented signs of telomere stress, as telomere intensity was 20% higher in the treated group than in vehicle-receiving mice. Telomere intensity sum is considered the best accurate and robust readout of telomere length because it represents the total amount of PNA probes bound along the telomere [44,74]. The development of telomere instability in the pancreatic islet nuclei of *Atrx^HOM^* mice ascertains the importance of Atrx in chromatin remodelling, but was not enough to precipitate tumour development.

We created and studied an *Atrx* GEMM that presents mild endocrine dysfunction and is insufficient to induce PanNET formation. Deleting Atrx from β cells may constitute a late and cumulative event solely. Therefore, a single KO event may not be enough to drive NE tumourigenesis in the pancreas. When comparing MEN1 to ATRX hereditary syndromes, the first is characterised by PanNET development. Still, the *ATRX* germ-line mutation in the second does not seem to hold a dominant role in NE tumourigenesis. More importantly, human PanNET epigenomes and transcriptomes are being increasingly explored based on the expression of aristaless-related homeobox (ARX) and pancreatic and duodenal homeobox 1 (PDX1), drivers of α- and β-cell differentiation, respectively. It was described that the subset of *ATRX*/*DAXX*-mutated PanNETs is *ARX*^+^/*PDX1*^-^, while β cells seem less susceptible to such mutations, as *ARX*^-^/*PDX1*^+^ cases do not harbour them [75,76].

Finally, whether *ATRX*/*DAXX* mutations found in human PanNETs are truly driver or passenger events remains to be clarified [17,18,19]. We believe that, in the short term, suitable GEMMs will be designed to ascertain the role of ATRX and DAXX in PanNET tumourigenesis either affecting β- or β-cell populations. Our model could be helpful for further metabolic studies or, instead, be modified with the aggravation of the endocrine disfunction or by even adding other genetic hits to evaluate tumourigenesis.

## 5. Conclusions

We report, for the first time, a GEMM of *Atrx* disruption in β cells and its insufficient capacity to induce PanNET formation. Instead, loss of Atrx at endocrine islets seems to play a role in anticipation and aggravation of inflammageing and ageing-related deterioration of endocrine functions. *Atrx^KO^* consistently presented higher weights and increased glycaemic levels than the age-matched controls. When submitted to ipGTT, these mice (especially the *Atrx^HOM^*) exhibited glucose intolerance with sexual dimorphism (detected earlier in males).

Unexpectedly, a compensatory increase of endocrine fraction and/or hyperinsulinaemia was not observed, suggesting the development of a mild dysfunction with which the basal metabolism of mice can still cope. We believe that our model has a dual utility for future studies, either exploring the uncovered role on endocrine dysfunction and/or exploring the putative synergic effect of additional genetic alterations towards tumourigenesis.

## Supplementary Materials

The following supporting information can be downloaded at: https://www.mdpi.com/article/10.3390/cancers14163865/s1, Supplementary File S1 (Materials and Methods): Figure S1: Schematic diagrams of Atrx and targeted deletion of exon 18; Figure S2—Age groups and age formula; Figure S3—White Blood Cell (WBC) scatter plots; Figure S4—Pipeline of telomere evaluation in Imaris; Table S1—Primers and genotyping conditions; Table S2—Study population and procedures; Table S3—List of antibodies used in immunohistochemistry; Table S4—Treatment scheme of BRACO-19 trial. Supplementary File S2 (Results): Figure S1—Pancreatic inflammatory lesions in 12–24 mo. age groups; Figure S2—Pancreatic inflammatory and ageing lesions by three age groups; Figure S3—Weights and glycaemias distribution using Orange Data Mining; Figure S4—Endocrine fraction, islet count, and mean islet are; Figure S5—Non-fasted and fasted insulinaemias; Figure S6—Intraperitoneal glucose tolerance tests of 3 mo.-old mice; Figure S7—Supplementary outcomes of BRACO-19 trial; Table S1—Overview of main results by age and genotype; Table S2—Tumour incidence analysis; Table S3—Hemograms, all parameters.
