# Supplementary material for: Characterisation of an Atrx Conditional Knockout Mouse Model: Atrx Loss Causes Endocrine Dysfunction Rather Than Pancreatic Neuroendocrine Tumour
Source: Cancers (Basel). 2022 Aug 10;14(16):3865. doi: 10.3390/cancers14163865 (PMC9406167; doi:10.3390/cancers14163865)
Supplement: Supplementary file 1 [file cancers-14-03865-s001.zip › 20220810-GasparTB2022_SupplementaryData.pdf]

Characterisation of an Atrx conditional knockout mouse model: Atrx loss causes endocrine dysfunction rather than pancreatic neuroendocrine tumour

Supplementary Data

Gaspar TB et al., 2022

Table of Contents

Supplementary File S1: Figure S1 – Schematic diagrams of Atrx and targeted deletion of exon 18 3

Supplementary File S1: Figure S2 – Age groups and age formula 4

Supplementary File S1: Figure S3 – White Blood Cell (WBC) scatter plots 5

Supplementary File S1: Figure S4 – Pipeline of telomere evaluation in Imaris 6

Supplementary File S1: Table S1 – Primers and genotyping conditions 7

Supplementary File S1: Table S2 – Study population and procedures 8

Supplementary File S1: Table S3 – List of antibodies used in immunohistochemistry 9

Supplementary File S1: Table S4 – Treatment scheme of BRACO−19 trial 10

Supplementary File S2: Figure S1 – Pancreatic inflammatory lesions in 12-24 mo. age groups .. 11

Supplementary File S2: Figure S2 – Pancreatic inflammatory and ageing lesions by three age groups 12

Supplementary File S2: Figure S3 – Weights and glycaemias distribution using Orange Data Mining 13

Supplementary File S2: Figure S4 – Endocrine fraction, islet count, and mean islet area 14

Supplementary File S2: Figure S5 – Non-fasted and fasted insulinaemias 15

Supplementary File S2: Figure S6 – Intraperitoneal glucose tolerance tests of 3 mo.-old mice .. 16

Supplementary File S2: Figure S7 – Supplementary outcomes of BRACO−19 trial 17

Supplementary File S2: Table S1 – Overview of main results by age and genotype 18

Supplementary File S2: Table S2 – Tumour incidence analysis 20

Supplementary File S2: Table S3 – Hemograms, all parameters 22

Supplementary File S1

Supplementary File S1: Figure S1 – Schematic diagrams of Atrx and targeted deletion of exon 18

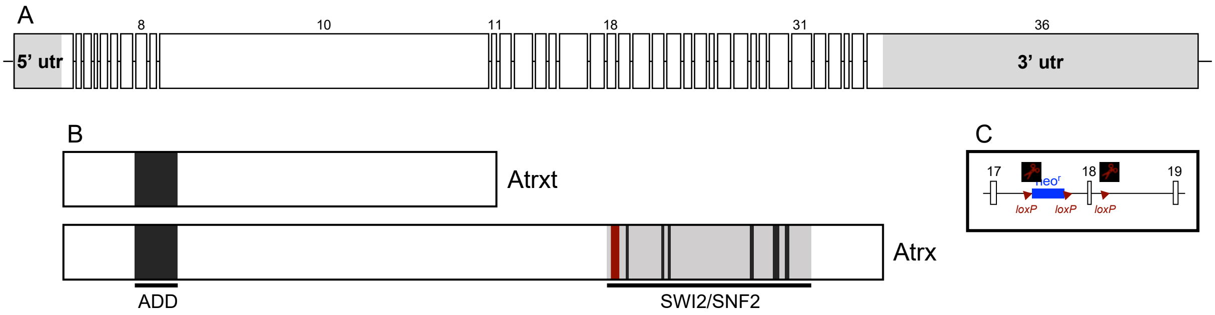

Figure S1 – Schematic diagrams of Atrx and targeted deletion of exon 18. A Atrx gene; boxes represent the 36 exons and horizontal lines represent the introns, which are not to scale; the 3' and 5' untranslated regions (**utr**) are shown flanking the open reading frame. **B** Atrx protein; representation of the truncated Atrx isoform (**Atrxt**) (200 kDa) and the full-length protein (**Atrx**) (280 kDa); Atrxt is produced when intron 11 is not spliced from the primary transcript, retaining the ADD domain but not the SWI2/SNF2 domain, whose seven motifs are illustrated; the motif painted in red is absent from *Atrx floxed* (*Atrx*^{KO}) mice. **C** strategy for targeted deletion of exon 18, using a *floxed* neo^{r} cassette inserted within intron 17 and *loxP* sites flanking exon 18; boxes represent the exons 17 to 19 and horizontal lines represent the respective introns; triangles represent the inserted loxP sites and scissors flank the total excised sequence. Figures adapted from Bérubé *et al.*, 2005, Garrick *et al.*, 2006 and Gibbons *et al.*, 2008.

Supplementary File S1: Figure S2 – Age groups and age formula

3 mo.	6 mo.	9 mo.	12 mo.	15 mo.	18 mo.	21 mo.	24 mo.
]1.50-4.49]	]4.50-7.49]	]7.50-10.49]	]10.50-13.49]	]13.50-16.49]	]16.50-19.49]	]19.50-22.49]	]22.50-25.49]

$$Age\ formula\ (in\ days) = \frac{DOD\ or\ DOP - DOB}{30}$$

Figure S2 – Age groups and age formula. DOB date of birth, **DOD** date of death, **DOP** date of procedure, **mo.** months. Animal ages were calculated using this formula and distribution. For most analyses, due to inferior number of animals, data collected at 9, 15 and 21 mo. were analysed together with data collected at 12, 18 and 21 mo., respectively.

Supplementary File S1: Figure S3 – White Blood Cell (WBC) scatter plots

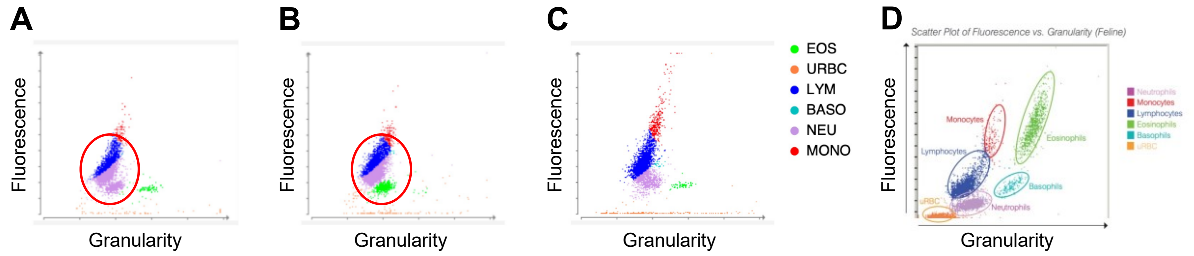

Figure S3 – White blood cell (WBC) scatter plots. BASO basophils, **EOS** eosinophils, **LYM** lymphocytes, **MONO** monocytes, **NEU** neutrophils, **URBC** unlysed red blood cells. **A** and **B** are examples of bad scatter plots (see inside red oval shape), with a wrong WBC differential count: decreased lymphocyte count (in blue) due to misclassification of neutrophils (in lilac), read as lymphopenia and neutrophilia (**A**), and neutrophil count replaced by eosinophil count (in green), read as lymphopenia, neutrophilia and eosinophilia (**B**); example of an accepted scatter plot of a good WBC differential count (**C**); example of an ideal WBC scatter plot, retrieved from IDEXX® ProCyte Dx Hematology Analyzer Operator's Guide (**D**).

Supplementary File S1: Figure S4 – Pipeline of telomere evaluation in Imaris

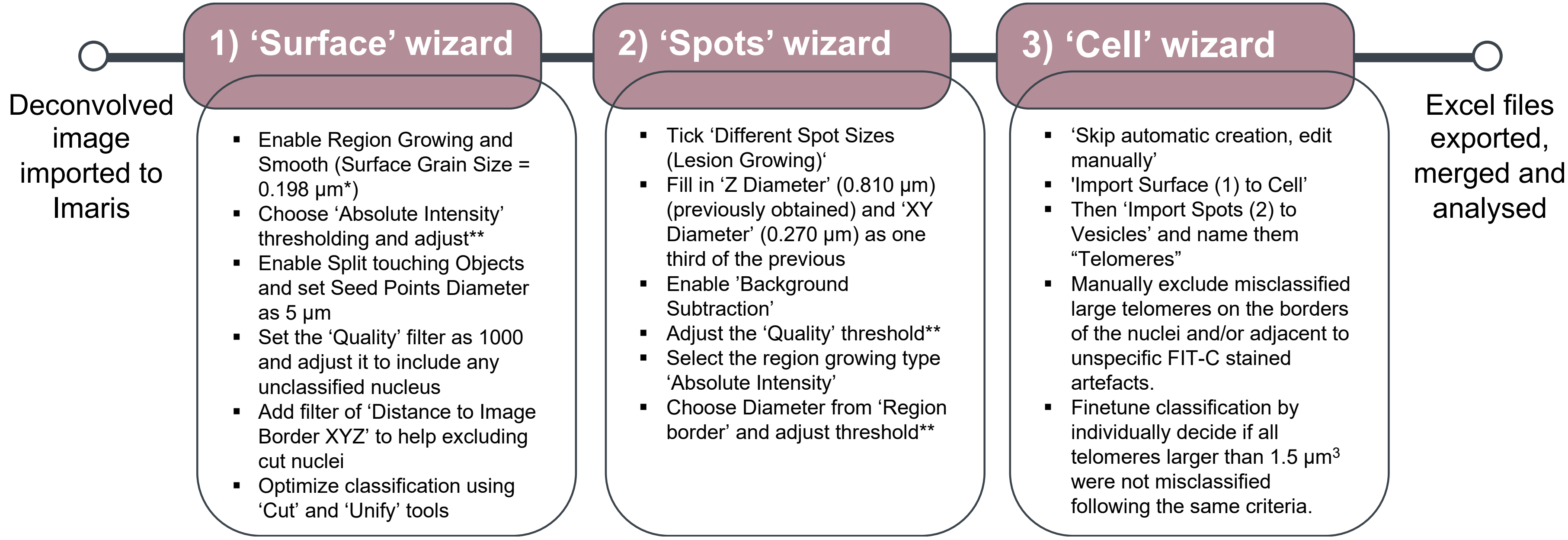

Figure S4 – Pipeline of telomere evaluation in Imaris. * calculated by Imaris, ** manually adjusted at approximately 1/e of the exponential decay. After deconvolution, images were imported to Imaris and the pipeline above was followed.

Supplementary File S1: Table S1 – Primers and genotyping conditions

Table S1 – Primers and genotyping conditions

Primers	Sequence (5’-3’)	T_a (º C)	Product length (bp)
**ATRX_int17_F**	GGAGAGGGAAGGAGGAAATG	60	KI = 199<br>WT =150
**ATRX_int17_R**	TAGCCATACCTGCAACCACA	60	
**OIMR1084_F**	GCGGTCTGGCAGTAAAAACTATC	60	KI = 100
**OIMR1085_R**	GTGAAACAGCATTGCTGTCACTT	60	
**N10_F**	GTATTGAATTGAAGCACCTTTGTTTGG	55	KI = 370<br>WT = 200
**N12_R**	CTGCCCAAGGCTCCCCCAG	55	
**Cre_F**	ATGTCCAATTTACTGACCGT	55	KI = 100
**Cre_R**	CGCCGCATAACCAGTGAAAC	55	

DNA Extraction: Samples were incubated at 56°C for 2 h on a thermal-shaker with 300 µL of lysis buffer [10 mM Tris (pH 7.5), 400 mM NaCl, 2 mM EDTA (pH 8.0)] (pH 7.3-7.5), 15 µL of 20% sodium dodecyl sulphate (SDS) Cat. No. 428018 (Merck), and 10 µL of 20 mg/mL proteinase K Ref. AM2548 (Ambion RNA by Life Technologies). Then, 100 µL of 6 M NaCl (saturated solution) was added to the extraction mixture, samples were mixed thoroughly by vortexing for 10 s, followed by centrifugation at 14000 rpm for 15 min to precipitate the residual cellular debris. The supernatant was transferred to a clean Eppendorf tube and 800 µL of 100% ethanol was added to each sample, mixed for 10 s, and centrifuged at 14000 rpm for 5 min to pellet the DNA. The DNA pellets were washed with 500 µL of 70% ethanol, followed by centrifugation at 14000 rpm for 5 min. The pellets were completely air dried and suspended in 80 µL of sterile nuclease-free water.

DNA Amplification: For conventional PCR the commercial master mix 2x My Taq HS Mix, Cat. No. Bio-25046 (Bioline) was used. PCR amplifications were carried out in the T-100 Thermal Cycler (Biorad). The reaction mixture was prepared in a 10 µL final volume containing 5 µL of master mix, 1-2 pmol of each primer and 1 µL of genomic DNA (~50-100 ng/µL). All assays included at least one positive control samples and a no-template control (containing all reaction components except the genomic DNA). The amplification protocol included an initial denaturation and enzyme activation at 95°C for 5 min followed by 30 cycles of denaturation at 95°C for 30 s, annealing at 56-60°C (according to each primer) for 90s, extension at 72°C for 30 s and a final extension at 60°C for 10 min.

Supplementary File S1: Table S2 – Study population and procedures

Table S2 – Study population and procedures

		Series 1 (2014-2018)			Series 2 (2018-2022)		
		$Atrx^{WT}$	***$Atrx^{HET}$***	***$Atrx^{HOM}$***	***$Atrx^{WT}$***	***$Atrx^{HET}$***	***$Atrx^{HOM}$***
	Euthanasias (n = 509) [a]	44 M<br>21 F	79 F	41 M<br>12 F	53 M<br>44 F	69 F	80 M<br>66 F
	HP evaluation of non-tumoural pancreas (n = 289) [b]	38 M<br>18 F	65 F	37 M<br>12 F	30 M<br>24 F	26 F	21 M<br>18 F
	HP evaluation of non-tumoural liver (n = 173) [c]	21 M<br>14 F	31 F	19 M<br>9 F	18 M<br>16 F	12 F	16 M<br>17 F
	IHC and HP characterisation of tumours (n = 42) [d]	10 M<br>3 F	10 F	11 M<br>0 F	3 M<br>1 F	0 F	2 M<br>2 F
	Endocrine fraction (n = 140) [e]	23 M<br>11 F	34 F	22 M<br>10 F	10 M<br>5 F	11 F	10 M<br>4 F
	ELISA (n = 83) [e]	5 M<br>4 F	5 F	10 M<br>3 F	11 M<br>7 F	11 F	14 M<br>15 F
**Longitudinal analyses***	**Weightings** (n = 898) [f]	-	-	-	76 M (n = 209)<br>55 F (n = 130)	100 F (n = 194)	73 M (n = 198)<br>43 F (n = 167)
	**Glycaemias** (n = 241) [f]	-	-	-	18 M (n = 53)<br>15 F (n = 35)	12 F (n = 42)	20 M (n = 57)<br>19 F (n = 54)
	**ipGTTs** (n = 65) [e]	-	-	-	12 M (n = 13)<br>9 F (n = 14)	7 F (n = 11)	16 M (n = 16)<br>9 F (n = 11)
	**Hemograms** (n = 94) [g]	-	-	-	11 M (n = 18)<br>9 F (n = 12)	12 F (n = 18)	14 M (n = 23)<br>12 F(n = 23)
	**BRACO−19** (n = 65) [h]	-	-	-	-	21 F	23 M<br>22 F
	**Tel-FISH** (n = 8) [i]	-	-	-	-	2 F	5 M<br>1 F

$Atrx^{WT}$ *$Atrx^{y/wt\ or\ wt/wt}$;Rip-$Cre^{+/-}$*, ***$Atrx^{HET}$*** *$Atrx^{f/wt}$;Rip-$Cre^{+/-}$*, ***$Atrx^{HOM}$*** *$Atrx^{y/f\ or\ f/f}$;Rip-$Cre^{+/-}$*, **F** female mice, **M** male mice. **HEP** humane endpoint, **HP** histopathological, **IHC** immunohistochemistry, **ipGTTs** intraperitoneal glucose tolerance tests, **Tel-FISH** telomere fluorescesce *in-situ* hybridisation. * number of measurements (**n**) are depicted in all genotypes, for both sexes. **Notes: a** euthanasias were either whenever HEP were reached (majority of series 1, and some mice in series 2) or predetermined (series 2); euthanasias occurred at all ages, and were then organised into eight age groups: 3, 6, 9, 12, 15, 18, 21, and 24 mo.; **b** total pancreas slides evaluated with HP score of pancreatic inflammation; **c** total liver slides evaluated with the nonalcoholic fatty liver disease (NAFLD) activity score (NAS); livers with evidence of hyperplasia or discrete benign tumor were also evaluated with NAS; **d** the 42 mice either bear benign or malignant tumours (n = 9 and n = 79, respectively); around 60% of the tumours were characterised with IHC; **e** performed in three age groups: 3, 6, and 12 mo.; **f** assessed at all ages in a longitudinal fashion, and then organised into five age groups (3, 6, 12, 18, and 24 mo.); measurements at T0 of ipGTT were also included; measurements of agitated mice were excluded; 347 and 84 animals were included in weight and glycaemia analyses, respectively; weights of mice by DOD were also included; **b-f** measurements at 9, 15, and 21 mo. were included in 12, 18 and 24 mo. age groups; **g** performed in four age groups: 3, 6, 12, and 18 mo. (most animals have more than one measurement over time); age group of 24 mo. included in age group of 18 mo.; **h** includes vehicles and treated mice from the three study groups (**see Supplementary File S1: Table S4**); **i** includes 8 mice from group III of BRACO−19 trial.

Supplementary File S1: Table S3 – List of antibodies used in immunohistochemistry

Table S3 – List of antibodies used in immunohistochemistry

Antigen	Species (clonality)	Source	Antibody (Reference)	RRID Code	Antigen retrieval (buffer, minutes)	Dilution (incubation time, hours)	Detection (DAB time, minutes)
**Chromogranin A**	Rabbit (P)	Synaptic Systems	259003	AB_2619972	Steamer (C, 40’)	1:400 (1)	2
**Synaptophysin**	Rabbit (M)	Thermo Fisher	9111-S0	AB_149939	Steamer (C, 40’)	1:100 (1)	2
**Insulin**	Mouse (P)	Zymed	18-0056	n.a.	Steamer (C, 40’)	1:200 (ON)	2
**Glucagon**	Rabbit (P)	Dako	N1541	n.a.	Steamer (C, 40’)	RTU (1)	2
**ATRX**	Rabbit (P)	Sigma-Aldrich	HPA001906	AB_1078249	Steamer (C, 40’)	1:500 (ON)	2
**Cre**	Rabbit (P)	Millipore	69050-3	AB_10806983	Steamer (C, 40’)	1:1000 (ON)	2
**Vimentin**	Rabbit (M)	Cell Signaling	5741	AB_10695459	Steamer (C, 40’)	1:250 (ON)	2
**Keratin, Pan**	Mouse (M)	Thermo Fisher	MS-343-P1	AB_61535	Water bath (E, 20’)	1:200 (1)	3
**CD45**	Rat (M)	BD Pharmingen	550539	AB_2174426	Microwave (E, 15’)	1:10 (ON)	8
**Ki67**	Rabbit (M)	Cell Marque	275R-18	AB_1158039	Pressure cooker (C, 65º C, 1’; 125º C, 10’)	1:100 (ON)	2
**MUM-1**	Rabbit (M)	Biocare Medical	901-352-082420	n.a.	Water bath (E, 20’)	1:50 (ON)	2
**CD31**	Mouse (M)	Dako	M0823	AB_2114471	Stove 37º C (Pe, 30) + freezer (TBS 1x, 3’)	1:50 (ON)	2
**CD18**	Mouse (M)	UC Davis School of Veterinary Medicine	n.a.*	n.a.	Water bath (C, 20’)	1:100 (ON)	2

M monoclonal, **P** polyclonal, **C** 1x citrate, **E** 1x EDTA, **ON** overnight (16-18 hr), **Pe** pepsin (1:50), **RTU** ready-to-use, *clone CA16.3C10.

Supplementary File S1: Table S4 – Treatment scheme of BRACO−19 trial

Table S4 – Treatment scheme of BRACO−19 trial

Age	1 mo.				2 mo.				3 mo.				4 mo.				5 mo.				6 mo.							
**Weeks**	1	2	3	4	5	6	7	8	9	10	11	12	13	14	15	16	17	18	19	20	21	22	23	24	25	**n**	**M**	**F**
**Group I** (n = 22)	Growing								**BRACO-19/** Vehicle				Eut													**16 B** 6 V	**5 B** 2 V	**11 B** 4 V
**Group II** (n = 23)	Growing								**BRACO-19/** Vehicle				Follow-up				Follow-up				Follow-up				Eut	**16 B** 7 V	**5 B** 3 V	**11 B** 4 V
**Group III** (n = 21)	Growing								**BRACO-19/** Vehicle				Follow-up				**BRACO-19/** Vehicle				Follow-up				Eut	**12 B** 9 V	**5 B** 3 V	**7 B** 6 V

B BRACO−19-treated mice, **V** vehicle-administered mice (dH_2O). A subcohort composed of 66 mice was enrolled in BRACO−19 trial; mice of Group **I** were treated for 4 weeks (weeks 9-12) (Monday to Friday) and euthanised after that period, with 3 mo. (week 13, Monday); mice of Group **II** were also treated for the same period, but were kept alive for 3 mo., and were euthanised with 6 mo. (week 25, Monday); mice of Group **III** received an additional treatment for another 4 weeks (weeks 17-20) and were kept alive for 1 mo., and euthanised also with 6 mo. (week 25, Monday). Composition of each group is depicted in the last three columns.

Supplementary File S2

Supplementary File S2: Figure S1 – Pancreatic inflammatory lesions in 12-24 mo. age groups

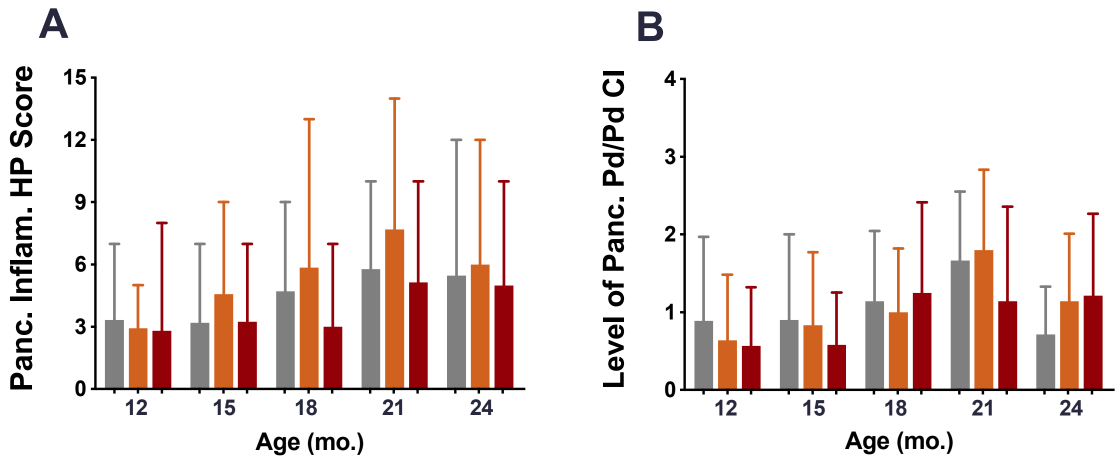

Figure S1 – Pancreatic inflammatory lesions in 12-24 mo. age groups. Pancreatic inflammation is higher in *Atrx*^{KO} mice, especially *Atrx*^{HET} females (**A**), mostly due to increased periductal/perivascular (Pd/Pv) chronic inflammation (CI) (**B**). **Grey** *Atrx*^{WT}, **red** *Atrx*^{HOM}, **orange** *Atrx*^{HET}. Results are shown as mean +/- range values (**A**) and mean +/- SD (**B**).

Supplementary File S2: Figure S2 – Pancreatic inflammatory and ageing lesions by three age groups

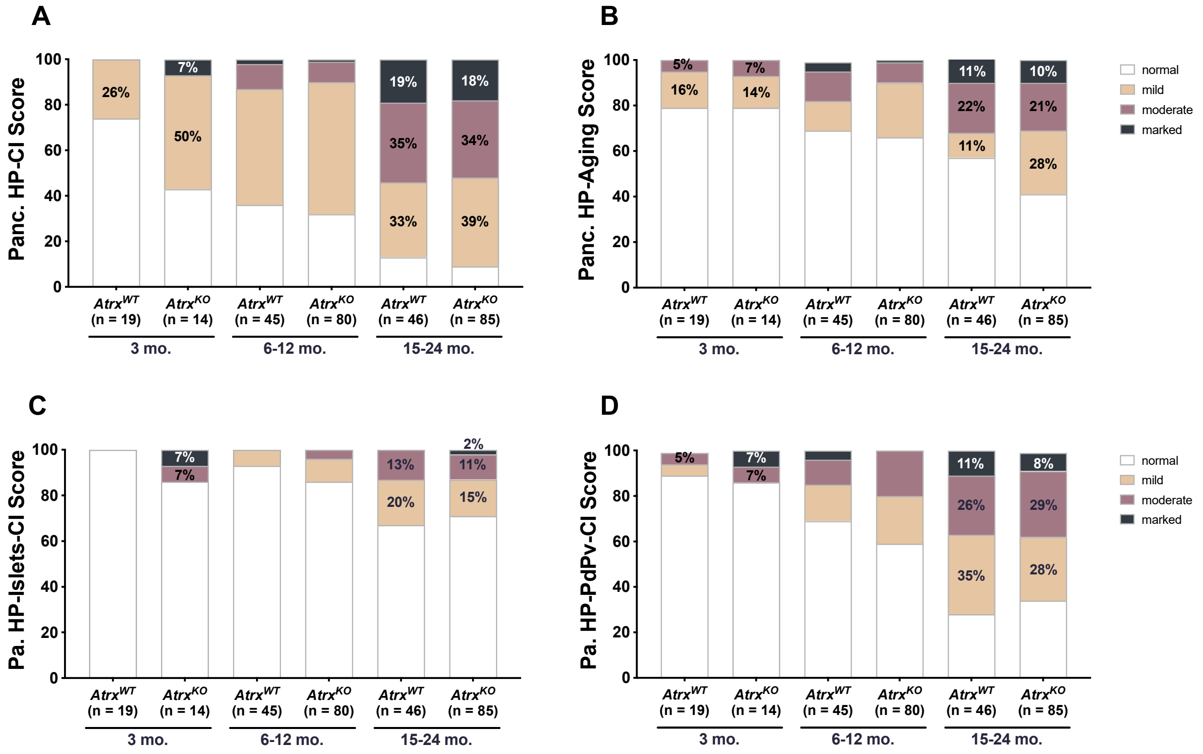

Figure S2 – Pancreatic inflammatory and ageing lesions by three age groups. Distribution by three age groups (3 mo., 6-12 mo., and 15-24 mo.) of pancreatic histopathological (HP) score of chronic inflammation (CI) divided in four intensity levels: normal (scores 0-1), mild (2-4), moderate (5-7), and marked (≥ 8) (**A**); pancreatic HP score of ageing parameters (including ductal/vascular dilation, focal acinary atrophy, and presence of ductal dysplasia) divided in four intensity levels normal (scores 0-1), mild (2), moderate (3), and marked (≥ 4) (**B**); and pancreatic HP score of CI at istet (**C**) and at periductal/perivascular (Pd/Pv) (**D**) locations, following a four-level scoring system (0 corresponds to absence of the alteration (< 5%), 1 to low-grade lesion (5-33% altered), 2 to moderate-grade lesion (33-66%), and 3 to high-grade lesion (> 66% altered)).

Supplementary File S2: Figure S3 – Weights and glycaemias distribution using Orange Data Mining

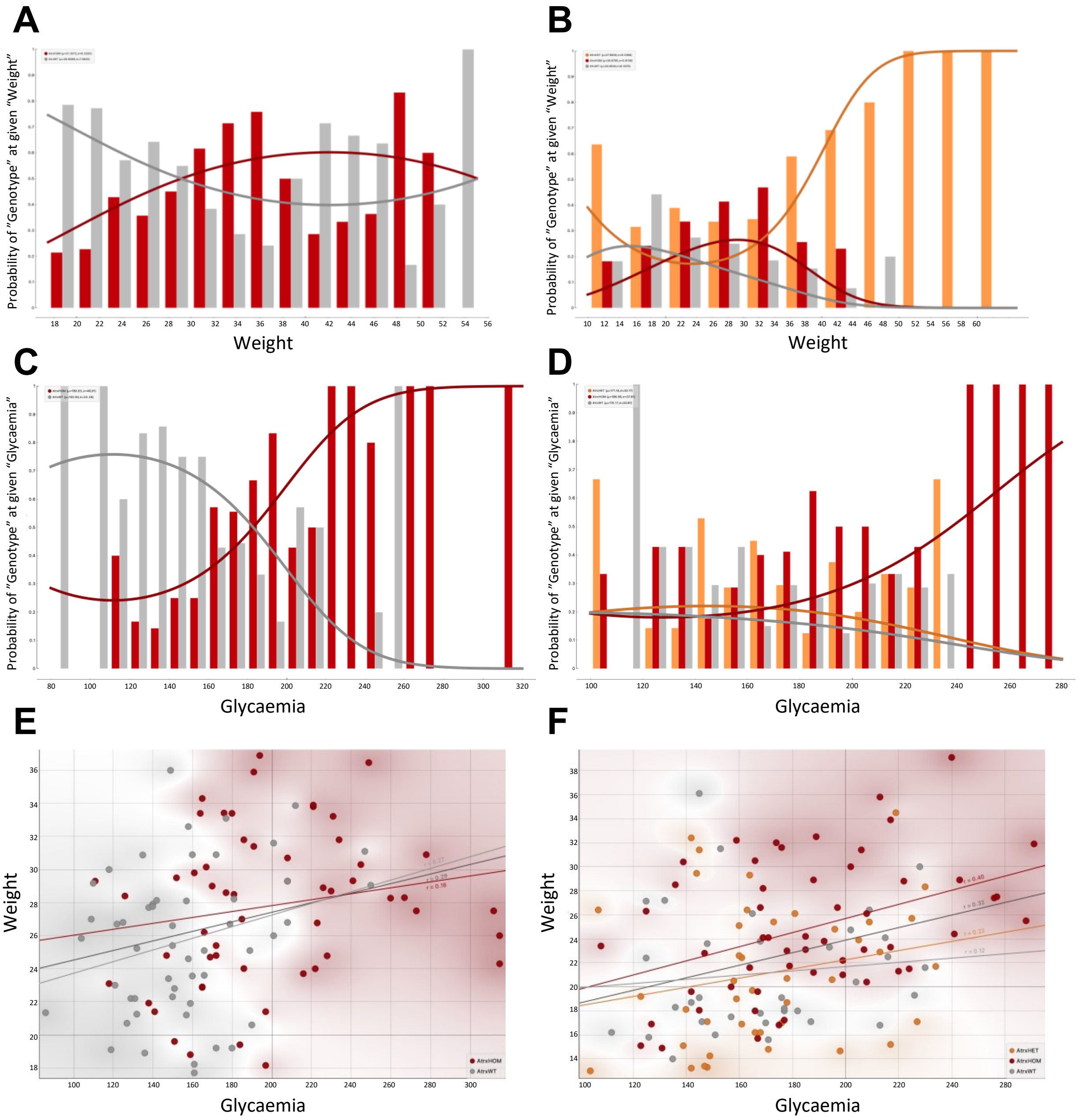

Figure S3 – Weights and glycaemias distribution using Orange Data Mining. Probability of genotype at a given weight of male (**A**) and female (**B**) mice. Distribution of the probability of each genotype at given glycaemia value of male (**C**) and female (**D**) mice. Scatter plots of glycaemia and weight in male (**E**) and female (**F**) mice; colour regions highlight the difference between *Atrx*^{WT} and *Atrx*^{HOM} male mice, as the later exhibit higher weights and higher glycaemias (**E**). **Grey** *Atrx*^{WT}, **red** *Atrx*^{HOM}, **orange** *Atrx*^{HET}.

Supplementary File S2: Figure S4 – Endocrine fraction, islet count, and mean islet area

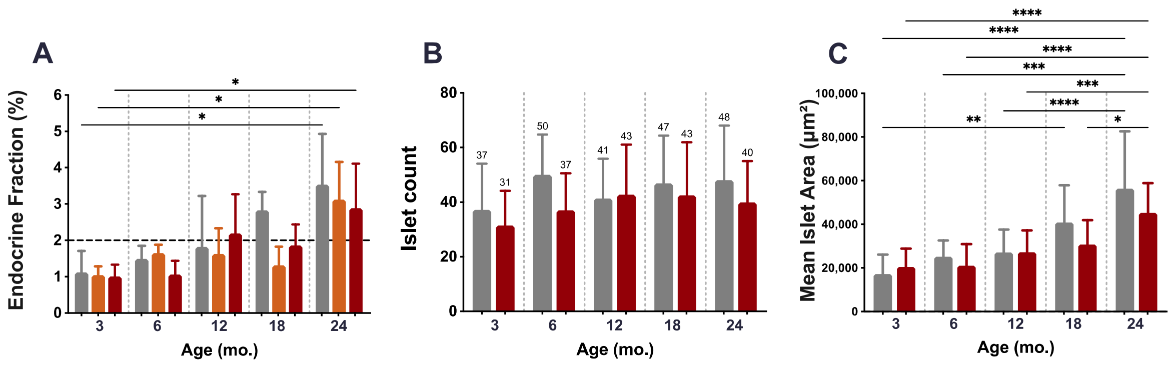

Figure S4 – Endocrine fraction, islet count, and mean islet area. Distribution by five age groups (3, 6, 12, 18, and 24 mo.) of endocrine fraction considering three genotype groups (**A**); islet number, obtained after manual counting in H&E-stained slide on optic microscope (n = 284) (**B**); mean islet area was obtained by the division of the obtained area in HALO and the respective manual count (**C**). **Grey** *Atrx*^{WT}, **red** *Atrx*^{HOM} (**A**) or *Atrx*^{KO} (**B, C**), **orange** *Atrx*^{HET}. Data is shown as median +/- IQR (**A**) and mean +/- SD (**B, C**).

Supplementary File S2: Figure S5 – Non-fasted and fasted insulinaemias

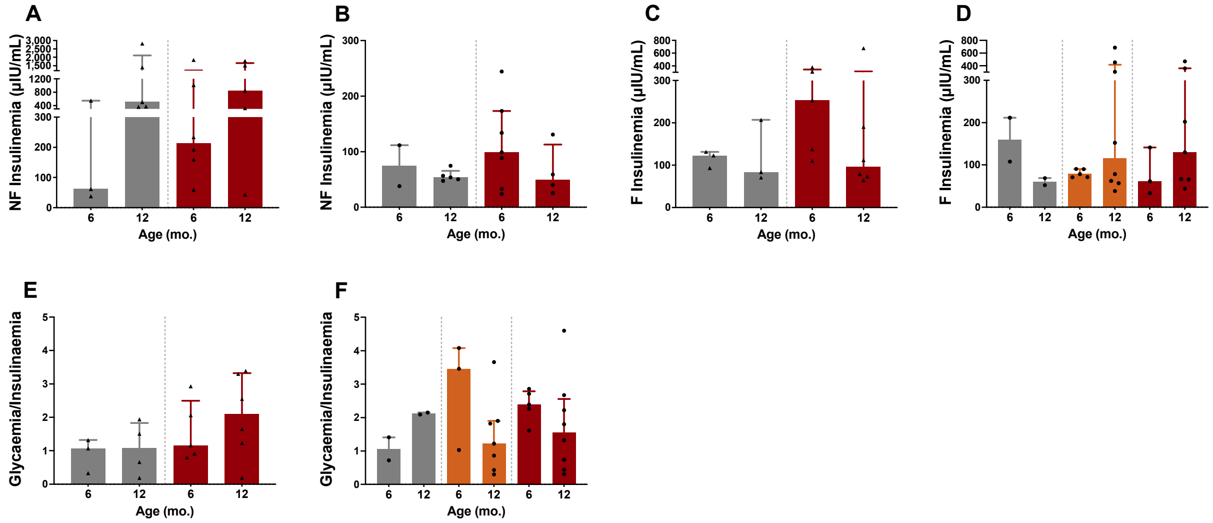

Figure S5 – Non-fasted and fasted insulinaemias. Non-fasted (NF) insulinaemia values were obtained from frozen serum collected by the time of euthanasia of male (**A**) and female (**B**) mice of Series 1. Insulin of NF *Atrx*^{HOM} mice (red bars) by 6 mo. is higher than age-matched controls (grey bars), especially in males; NF insulinaemia seems to increase with ageing in male mice of both genotypes, but not in females. In graph B, values of *Atrx*^{HET} and *Atrx*^{HOM} genotypes were plotted together due to lack of values of 6-mo. *Atrx*^{HOM} females. Fasted (F) insulinaemia values were obtained from 6-hour fasted male (**C**) and female (**D**) mice of Series 2. *Atrx*^{HOM} male mice exhibit increased F insulinaemia by 6 mo. than age-matched controls; median insulinaemia values seem to increase with ageing in *Atrx*^{HET} and *Atrx*^{HOM} females but to decrease in males. However, there are clusters of values of higher insulinaemias in *Atrx*^{KO} males and females. The 6-hour fasted glycamia/insulinaemia ratios also indicate that *Atrx*^{KO} have a tendency for higher values (**E, F**). **Grey** *Atrx*^{WT}, **red** *Atrx*^{HOM}, **orange** *Atrx*^{HET}. Triangles and circles represent values of male and female mice, respectively. All values are represented as median +/- IQR.

Supplementary File S2: Figure S6 – Intraperitoneal glucose tolerance tests of 3 mo.-old mice

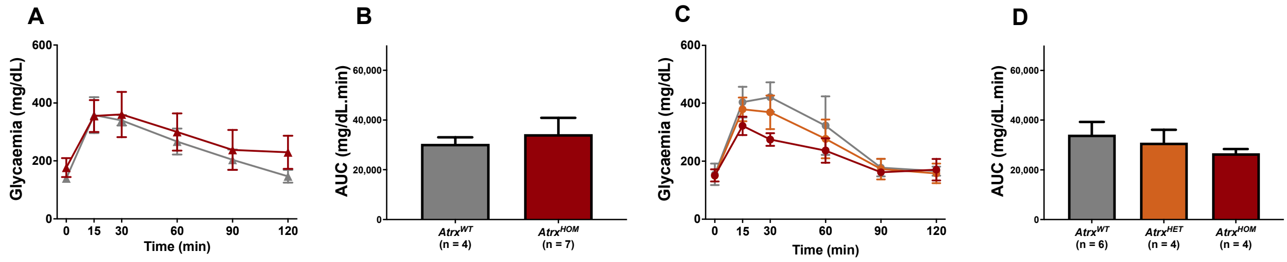

Figure S6 – Intraperitoneal glucose tolerance tests of 3 mo.-old mice. Intraperitoneal glucose tolerance tests of 3 mo.-old male (**A**, **B**) and female mice (**C**, **D**). **B** and **D** are the respective areas under the curve (AUC). **Grey** *Atrx*^{WT}, **red** *Atrx*^{HOM}, **orange** *Atrx*^{HET}.

Supplementary File S2: Figure S7 – Supplementary outcomes of BRACO−19 trial

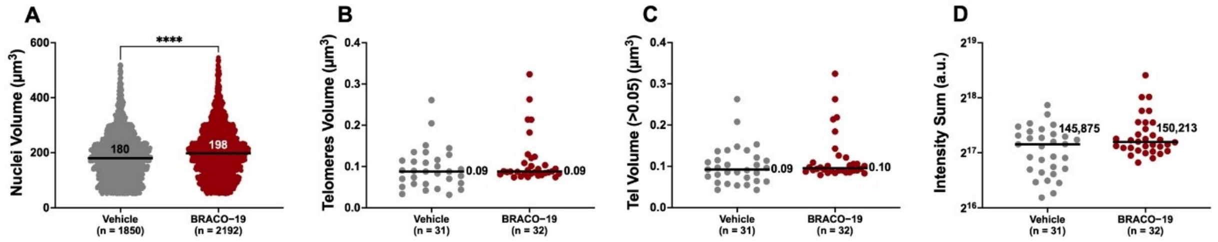

Figure S7 – Supplementary outcomes of BRACO−19 trial. a.u. arbitrary units. Pancreatic islet cell nuclei are significantly bigger in treated group (**A**). Average telomere volume (**B, C**) and intensity sum (**D**) per analysed image follow the same tendency as raw data analysis (**see Figure 7**) but shows no statistical significance. Data of four graphs did not pass the normality tests, Mann-Whitney tests were then performed, and median values presented. **** $p < 0.0001$. A Log 2 scale and a Power of 2 tick format were used for the representation of the intensity sum (**D**). **Grey** *Atrx*^{WT}, **red** *Atrx*^{KO}.

Supplementary File S2: Table S1 – Overview of main results by age and genotype

Table S1 – Overview of main results by age and genotype

		3 mo.				6 mo.				9 mo.				12 mo.			
		Mean	Stdev	n	P	Mean	Stdev	n	P	Mean	Stdev	n	P	Mean	Stdev	n	P
Weights	M and F *Atrx*^{WT}	20,09	3,55	74	a	24,13	4,50	57	a, e, i	27,05	5,41	46	e, k, m	29,77	5,92	50	k
	M and F *Atrx*^{HOM}	20,92	3,82	55	b, #/*	26,37	3,88	64	b, f, i, #/*	30,23	3,91	51	f, m	30,88	5,14	63	#
	F *Atrx*^{HOM}	19,32	3,33	31	c	25,30	3,96	32	c, g, j	28,55	3,81	22	g, n	29,00	4,20	28	
	F *Atrx*^{HET}	17,77	4,23	26	d	22,44	4,25	44	d, h, j	25,37	4,89	23	h, l, n	31,16	8,66	32	l
	Correlations	-				-				-				-			
Glycaemias	M and F *Atrx*^{WT}	154,50	33,17	28	b	165,44	28,18	28	c					169,38	41,54	16	e
	M and F *Atrx*^{HOM}	178,42	35,22	33	a, b, #	211,00	48,09	26	a, c, #					203,44	38,75	27	d, e, *
	F *Atrx*^{HOM}	176,36	37,07	14		194,57	34,49	14						196,23	29,45	13	f
	F *Atrx*^{HET}	164,50	30,91	16		184,38	34,47	13						169,00	21,28	6	f
	Correlations	# Pearson's r = 0.531 ▲				# Pearson's r = 0.423 ▲								-			
GTT-AUC	M and F *Atrx*^{WT}	32670,20	4514,00	10		34503,30	8539,88	10	b					31256,00	6994,69	7	c
	M and F *Atrx*^{HOM}	31551,36	6438,30	11	a	51635,63	10830,02	8	a, b					51957,50	9267,16	8	c
	F *Atrx*^{HOM}	26668,25	1707,98	4		46785,00	17119,34	3						49134,75	7085,76	4	
	F *Atrx*^{HET}	30948,75	5195,00	4		39641,50	6674,58	4						39327,67	13022,75	3	
	Correlations	-				-								-			
EF	M and F *Atrx*^{WT}	1,22	0,52	10		1,49	0,40	5						1,96	0,98	14	
	M and F *Atrx*^{HOM}	1,00	0,33	8		1,67	1,77	8	*					2,10	1,16	13	#
	F *Atrx*^{HOM}	1,15	0,13	4		1,26	0,41	3						1,43	0,63	5	
	F *Atrx*^{HET}	1,09	0,23	5		1,54	0,75	7						1,58	0,77	10	
	Correlations	-				* Kendall's τ = 0.714 ▲▲								# Kendall's τ = 0.604 ▲			
HP-CI-Pa	M and F *Atrx*^{WT}	1,11	0,99	19		1,60	1,07	10		1,94	1,88	16	a	3,68	2,47	19	a
	M and F *Atrx*^{HOM}	1,75	2,66	8	*	1,20	0,92	10		2,43	1,99	7		2,81	1,89	21	
	F *Atrx*^{HOM}	2,25	3,86	4		1,33	0,58	3		4,00	n.a.	1		2,33	1,50	9	
	F *Atrx*^{HET}	2,33	1,51	6		2,52	1,29	21		3,33	2,42	6		2,93	1,53	15	
	Correlations	* Kendall's τ = 0.791 ▲▲				-								-			
HP-NAS-Li	M and F *Atrx*^{WT}	3,18	1,72	11		4,40	1,52	5	b	5,56	0,88	9		6,40	2,59	15	
	M and F *Atrx*^{HOM}	3,14	1,86	7	a	6,13	2,10	8	a, b	5,00	2,83	2		7,69	1,82	16	
	F *Atrx*^{HOM}	3,00	0,00	3		6,67	0,58	3		3,00	n.a.	1		6,63	1,19	8	c
	F *Atrx*^{HET}	3,50	1,73	4		5,20	1,48	5		5,33	1,53	3		6,33	1,94	9	
	Correlations	-				-								-			
WBC	M and F *Atrx*^{WT}	11,03	2,37	11	a	10,39	2,59	8	b					9,30	2,09	5	
	M and F *Atrx*^{HOM}	8,25	2,82	14	a	7,76	2,53	14	b					9,42	3,20	10	
	F *Atrx*^{HOM}	7,39	2,27	7		6,70	2,80	7						8,95	3,90	6	
	F *Atrx*^{HET}	10,90	3,68	5		9,13	3,77	6						8,05	2,62	2	
	Correlations	-				-								-			
%Lym	M and F *Atrx*^{WT}	78,18	8,96	12		82,61	6,77	8						75,28	11,19	5	
	M and F *Atrx*^{HOM}	86,10	1,68	15		81,41	5,15	14						76,34	8,70	10	c, *
	F *Atrx*^{HOM}	85,93	1,42	7		79,24	5,00	7						77,13	7,52	6	d
	F *Atrx*^{HET}	87,15	1,30	4	b	67,10	12,54	6	b, f					81,50	7,92	2	
	Correlations	-				-								* Kendall's τ = -0.867 ▼▼			

		15 mo.				18 mo.				21 mo.				24 mo.				
		Mean	Stdev	n	P	Mean	Stdev	n	P	Mean	Stdev	n	P	Mean	Stdev	n	P	total n
Weights	M and F *Atrx*^{WT}	28,37	4,80	36	o	28,34	4,35	29	p	32,96	9,31	29	p, #	33,33	5,69	18		339
	M and F *Atrx*^{HOM}	30,45	4,57	54	o, #	30,62	5,38	40	#	32,52	5,51	20		36,33	7,60	18		365
	F *Atrx*^{HOM}	28,52	5,15	26		29,36	6,49	21		31,53	7,85	5		38,02	1,15	2		167
	F *Atrx*^{HET}	31,29	6,40	21		31,87	4,21	13	q	39,33	10,55	17	q	35,75	9,51	18		194
	Correlations	-				-				-				-				**898**
Glycaemias	M and F *Atrx*^{WT}					156,43	24,72	7						157,22	44,52	9		88
	M and F *Atrx*^{HOM}					181,90	49,40	20	d					184,20	31,51	5		111
	F *Atrx*^{HOM}					172,40	52,55	10						193,00	40,73	3		54
	F *Atrx*^{HET}					182,75	37,13	4						138,00	28,48	3		42
	Correlations					-								-				**241**
GTT-AUC	M and F *Atrx*^{WT}																	27
	M and F *Atrx*^{HOM}																	27
	F *Atrx*^{HOM}																	11
	F *Atrx*^{HET}																	11
	Correlations																	**65**
EF	M and F *Atrx*^{WT}					3,06	1,48	9						3,28	1,56	11		49
	M and F *Atrx*^{HOM}					2,14	0,63	8						2,88	1,22	9		46
	F *Atrx*^{HOM}					1,29	0,40	2						n.a.	n.a.	n.a.		14
	F *Atrx*^{HET}					1,65	0,76	11	a					3,23	0,99	12	a	45
	Correlations					-								-				**140**
HP-CI-Pa	M and F *Atrx*^{WT}	3,20	2,10	10		4,71	3,20	7		5,79	2,42	14		5,47	3,07	15		110
	M and F *Atrx*^{HOM}	3,25	1,60	12		3,00	2,29	9		5,14	3,44	7		5,00	2,69	14		88
	F *Atrx*^{HOM}	3,40	1,14	5		3,00	2,35	5		7,00	2,83	2		4,00	n.a.	1		30
	F *Atrx*^{HET}	4,58	2,91	12		5,86	3,58	7		7,70	3,33	10		6,00	3,19	14		91
	Correlations					-								-				**289**
HP-NAS-Li	M and F *Atrx*^{WT}	6,50	3,02	6		7,67	2,88	6		7,40	2,72	10	#	5,57	1,51	7	e	69
	M and F *Atrx*^{HOM}	5,71	2,50	7	#	5,00	2,39	8		5,67	1,51	6	d	9,29	1,80	7	d, e	61
	F *Atrx*^{HOM}	4,00	2,00	3	c	4,60	2,88	5		6,50	0,71	2		9,00	n.a.	1		26
	F *Atrx*^{HET}	7,43	2,70	7		6,83	2,32	6		8,67	3,14	6		6,67	1,53	3		43
	Correlations	#15 Kendall's τ = 0.781 ▲▲								# Kendall's τ = 0.736 ▲▲				-				**173**
WBC	M and F *Atrx*^{WT}					11,15	0,77	4	c									28
	M and F *Atrx*^{HOM}					8,55	1,57	6	c									44
	F *Atrx*^{HOM}					7,77	1,76	3										23
	F *Atrx*^{HET}					7,00	1,85	4										17
	Correlations					-												**89**
%Lym	M and F *Atrx*^{WT}					73,44	14,29	5										30
	M and F *Atrx*^{HOM}					58,88	5,51	6	c, #									45
	F *Atrx*^{HOM}					62,63	4,92	3	d									23
	F *Atrx*^{HET}					70,90	4,69	4										16
	Correlations					# Kendall's τ = 0.733 ▲▲												**91**

Overview of the results of eight parameters distributed by **eight age groups** (3 to 24 months (mo.)) and by **four genotype groups** (male and female *Atrx*^{WT}, male and female *Atrx*^{HOM}, female *Atrx*^{HOM}, and female *Atrx*^{HET}). For each parameter, statistical comparisons were performed to assess **age-related changes** – over time (3 vs 6 mo., 6 vs 9 mo., 9 vs 12 mo., 12 vs 15 mo., 15 vs 18 mo., 18 vs 21 mo., and 21 vs 24 mo.), for each genotype group – and to assess **genotype-related changes** – within each age group, among genotypes (*Atrx*^{WT} *vs Atrx*^{HOM} of both sexes, and *Atrx*^{HET} vs *Atrx*^{HOM} females). Correlations were assessed all pairs of parameters, between *Atrx*^{WT} *vs Atrx*^{HOM} of both sexes; **#/✱** indicate correlation pairs (read vertically), **triangles** indicate correlation strength and direction; whenever both genotypes presented statistically significant correlations ($p < 0.05$), only the largest was considered. Data is presented as mean (**$\bar{x}$**) and standard deviation (**SD**) (weights and glycaemias) or median (**M**) and interquartile range (**IQR**) (all the remaining parameters), count (**n**) and P-value (**P**); a red-yellow-green colour scheme was used based on the mean/median values (red represent the highest value); all *p*-values < 0.05 are represented by pairs of lowercase letters – **same letter** represent statistically significant difference within each parameter. Independent t-tests and Pearson's correlation was performed in larger groups (n ≥ 15), while Mann-Whitney tests and Kendall's nonparametric tests were performed in smaller groups (n < 15). **GTT-AUC** glucose tolerance test's area under the curve, **EF** endocrine fraction, **HP-CI-Pa** histopathological evaluation of chronic inflammation (CI) in pancreas slides (Pa), **HP-NAS-Li** histopathological evaluation of non-alcoholic fatty liver disease activity score (NAS) in liver slides (Li), **WBC** white blood cell count, **%Lym** lymphocyte percentage.

Supplementary File S2: Table S2 – Tumour incidence analysis

Table S2 – Tumour incidence analysis

Tissue	Tumour type	$Atrx^{WT}$	$Atrx^{HET}$	$Atrx^{HOM}$
	Total mice with malignant tumour (median age, mo.) (n = 42)	13 M (22)<br>4 F (26)	10 F (19)	13 M (22)<br>2 F (24)
	Total mice euthanised mice ≥ 18 mo.[a] (% with malignant tumour) (n = 125)	29 M (45)<br>14 F (29)	40 F (25)	29 M (45)<br>13 F (15)
**Tissue**	**Tumour type (n = 79)[b]**			
**Pancreas** (n = 19)	**Epithelial [4/19 (21)]**	**2/4 (50)**	**1/4 (25)**	**1/4 (25)**
	Carcinoma, highly undifferentiated	2/2 (100)		
	Hepatocellular carcinoma, mets.			1/1 (100)
	PDAC		1/1 (100)	
	**Mesenchymal [6/19 (32)]**	**1/6 (17)**	**4/6 (66)**	**1/6 (17)**
	Endothelial tumour		1/4 (25)	
	Hystiocytic sarcoma	1/1 (100)	1/4 (25)	
	Mesenchymal tumour, NOS		2/4 (50)	1/1 (100)
	**Lymphoma [8/19 (42)]**	**4/8 (50)**	**2/8 (25)**	**2/8 (25)**
	Lymphoma of the small bowel, mets.	1/4 (25)		
	Lymphoma, NOS	3/4 (75)	2/2 (100)	2/2 (100)
	**Other [1/19 (5)]**			**1/1 (100)**
	Carcinosarcoma			1/1 (100)
	Tumour incidence per genotype[c]	**57% L** > 29% E > 14% M		**41% M** > 33% L > 17% E > 9% O
**Lung** (n = 16)	**Epithelial [11/16 (69)]**	**4/11 (36)**	**1/11 (9)**	**6/11 (55)**
	Carcinoma, highly undifferentiated	1/4 (25)		
	Lung carcinoma, NOS	3/4 (75)		5/6 (83)
	Hepatocellular carcinoma, mets.			1/6 (17)
	PDAC, mets.		1/1 (100)	
	**Mesenchymal [2/16 (12)]**	**1/2 (50)**	**1/2 (50)**	
	Splenic hemangiosarcoma, mets.	1/1 (100)		
	Mesenchymal tumour, NOS		1/1 (100)	
	**Lymphoma [3/16 (19)]**	**2/3 (67)**		**1/3 (33)**
	Lymphoma of the small bowel, mets.	1/2 (50)		
	Lymphoma, NOS	1/2 (50)		1/1 (100)
	Tumour incidence per genotype[c]	**57% E** > 29% L > 14% M		**78% E** > 11% M/L
**Liver** (n = 14)	**Epithelial [8/14 (58)]**	**3/8 ()**	**1/8 ()**	**4/8 (50)**
	Hepatocellular carcinoma, clear cell	1/3 (33)		1/4 (25)
	Hepatocellular carcinoma, well-diff.	2/3 (67)		2/4 (50)
	Lung carcinoma, NOS, mets.			1/4 (25)
	PDAC, mets.		1/1 (100)	
	**Mesenchymal [3/14 (21)]**	**2/3 (67)**	**1/3 (33)**	
	Histiocytic sarcoma	1/2 (50)	1/1 (100)	
	Mesenchymal tumour, NOS	1/2 (50)		
	**Lymphoma [2/14 (14)]**		**1/2 (50)**	**1/2 (50)**
	Lymphoma, NOS		1/1 (100)	1/1 (100)
	**Other [1/14 (7)]**			**1/1 (100)**
	Hepatoblastoma			1/1 (100)
	Tumour incidence per genotype[c]	**60% E** > 40% M		**56% E** >22% L > 11% M/O
**Spleen** (n = 11)	**Epithelial [3/11 (27)]**	**1/3 (33)**	**1/3 (33)**	**1/3 (33)**
	Carcinoma, highly undifferentiated	1/1 (100)		
	Hepatocellular carcinoma, mets.			1/1 (100)
	PDAC, mets.		1/1 (100)	
	**Mesenchymal [6/11 (55)]**	**3/6 (50)**	**3/6 (50)**	

	Histiocytic sarcoma	2/3 (67)	1/3 (33)	
	Mesenchymal tumour, NOS		2/3 (67)	
	Splenic hemangiosarcoma	1/3 (33)		
	**Lymphoma [2/11 (18)]**	**1/2 (50)**	**1/2 (50)**	
	Lymphoma, NOS	1/1 (100)	1/1 (100)	
**L.N./ Mesentery** (n = 8)	**Epithelial [4/8 (50)]**	**2/4 (50)**	**1/4 (25)**	**1/4 (25)**
	Carcinoma, highly undifferentatied	2/2 (100)		
	Hepatocellular carcinoma, mets.			1/1 (100)
	PDAC, mets.		1/1 (100)	
	**Mesenchymal [4/8 (50)]**		**4/4 (100)**	
	Endothelial tumour, NOS		2/4 (50)	
	Histiocytic sarcoma		2/4 (50	
**Kidney** (n = 3)	**Epithelial [1/3 (33)]**	**1/1 (100)**		
	Carcinoma, highly undifferentiated	1/1 (100)		
	**Mesenchymal [1/3 (33)]**			**1/1 (100)**
	Mesenchymal tumour, NOS			1/1 (100)
	**Lymphoma [1/3 (33)]**		**1/1 (100)**	
	Lymphoma, NOS		1/1 (100)	
**Small bowel** (n = 3)	**Epithelial [1/3 (25)]**	**1/1 (100)**		
	Jejunal carcinoma, NOS	1/1 (100)		
	**Mesenchymal [1/3 (25)]**		**1/1 (100)**	
	Mesenchymal tumour, NOS		1/1 (100)	
	**Lymphoma [1/3 (25)]**			**1/1 (100)**
	Lymphoma of the small bowel, NOS			1/1 (100)
**Unknown** (n = 3)	**Mesenchymal [3/3 (100)]**	**1/3 (33)**	**2/3 (67)**	
	Endothelial tumour, NOS	1/1 (100)		
	Histiocytic sarcoma		1/2 (50)	
	Mesenchymal tumour, NOS		1/2 (50)	
**Ovary** (n = 1)	**Epithelial [1/1 (100)]**		**1/1 (100)**	
	Ovarian carcinoma, NOS		1/1 (100)	
**Uterus** (n = 1)	**Mesenchymal [1/1 (100)]**		**1/1 (100)**	
	Mesenchymal tumour, NOS		1/1 (100)	
**Total** (n = 79)	*Tumour incidence per genotype*[c]	**45% E** > 28% M/L	**40% E** > 38% M > 18% L > 4% O	

a includes animals belonging to age groups of 18, 21 and 24 mo., **b** includes 79 malignant tumours; benign tumours (n = 9) include lipoma (n = 2, 1 in *Atrx*^{WT}, 1 in *Atrx*^{HOM}), hepatic hemangioma (n = 1, *Atrx*^{HOM}) and hepatocellular adenoma (n = 6, 3 in *Atrx*^{WT}, 3 in *Atrx*^{HOM}), **c** the indicidence (%) was calculated in the three most common tumour locations, for epithelial (**E**), mesenchymal (**M**), lymphoma (**L**), and other (**O**) tumours, considering the number of mice developing malignant tumours (n = 42); males and females were analised together; *Atrx*^{HET} and *Atrx*^{HOM} were analised together as *Atrx*^{KO}. **NOS** non-otherwise specified.

Supplementary File S2: Table S3 – Hemograms, all parameters

Table S3 – Hemograms, all parameters

	3 mo.		Total	6 mo.		Total	12 mo.		Total
**Values**	***Atrx*** ^{KO}	***Atrx*** ^{WT}		***Atrx*** ^{KO}	***Atrx*** ^{WT}		***Atrx*** ^{KO}	***Atrx*** ^{WT}	
Average of RBC	8.4	9.3	8.6	8.7	8.6	8.7	9.0	9.7	9.0
StdDev of RBC	0.8	0.8	0.9	0.8	0.1	0.7	0.7		0.7
Count of RBC	***13***	***6***	***19***	***13***	***4***	***17***	***8***	***1***	***9***
Min. of RBC	7.1	8.5	7.1	7.2	8.4	7.2	7.8	9.7	7.8
Max. of RBC	9.6	10.7	10.7	10.6	8.7	10.6	9.6	9.7	9.7
Average of % Ret	3.8	3.5	3.7	4.1	4.2	4.1	4.8	4.1	4.7
StdDev of % Ret	0.5	1.1	0.8	1.3	0.2	1.2	1.1		1.0
Count of % Ret	***7***	***7***	***14***	***11***	***3***	***14***	***6***	***1***	***7***
Average of Ret	308.6	282.2	295.4	353.6	356.8	354.3	424.7	399.9	421.1
StdDev of Ret	30.9	141.6	99.4	107.8	18.8	94.9	112.5		103.1
Count of Ret	***7***	***7***	***14***	***11***	***3***	***14***	***6***	***1***	***7***
Average of WBC	8.9	10.8	9.5	7.8	10.3	8.4	8.7	7.8	8.6
StdDev of WBC	3.3	3.1	3.3	3.4	3.1	3.4	3.5		3.3
Count of WBC	***13***	***7***	***20***	***13***	***4***	***17***	***8***	***1***	***9***
Min. of WBC	4.9	5.8	4.9	4.7	8.5	4.7	5.2	7.8	5.2
Max. of WBC	15.4	15	15.4	15.8	14.9	15.8	15.6	7.8	15.6
Average of % Neu	11.5	14.7	12.6	16.3	14.1	15.8	16.9	14.8	16.7
StdDev of % Neu	1.5	8.0	4.9	5.4	4.1	5.1	6.6		6.2
Count of % Neu	***13***	***7***	***20***	***13***	***4***	***17***	***8***	***1***	***9***
Min. of % Neu	9.0	6.9	6.9	7.3	10.1	7.3	7.3	14.8	7.3
Max. of % Neu	13.9	29.7	29.7	28.4	19.8	28.4	27.7	14.8	27.7
Average of % Lym	86.4	73.8	81.5	73.6	82.1	75.6	78.2	80.1	78.4
StdDev of % Lym	1.4	6.1	7.3	10.9	4.6	10.3	7.3		6.9
Count of % Lym	***13***	***7***	***20***	***13***	***4***	***17***	***8***	***1***	***9***
Min. of % Lym	83.4	67.5	67.5	50.9	75.8	50.9	68.6	80.1	68.6
Max. of % Lym	88.6	84.7	88.6	88.0	86.8	88.0	88.5	80.1	88.5
Average of Neu	1.0	1.1	1.0	1.3	1.5	1.3	1.4	1.2	1.3
StdDev of Neu	0.4	0.2	0.3	0.6	0.5	0.6	0.5		0.5
Count of Neu	***13***	***7***	***20***	***13***	***4***	***17***	***8***	***1***	***9***
Average of Lym	7.1	8.1	7.4	5.6	8.4	6.3	7.0	6.3	6.9
StdDev of Lym	2.2	2.3	2.2	2.1	2.5	2.5	3.3		3.1
Count of Lym	***13***	***7***	***20***	***13***	***4***	***17***	***8***	***1***	***9***
Average of PLTs	508.2	535.9	519.6	576.3	512.7	563.6	510.7	744.0	544
StdDev of PLTs	129.7	137.4	129.4	104.6	112.5	105.3	154.5		166.3
Count of PLTs	***10***	***7***	***17***	***13***	***3***	***16***	***6***	***1***	***7***
Average of NLR	14.2	18.5	15.5	22.8	17.4	21.5	22.5	18.4	22
StdDev of NLR	3.1	11.9	7.1	8.8	6.2	8.4	10.4		9.8
Count of NLR	***13***	***7***	***20***	***13***	***4***	***17***	***8***	***1***	***9***
Average of PLR	9301.4	7383.8	8582.3	11105.7	6112.3	10169.4	8154.4	11904.0	8690.1
StdDev of PLR	3825.7	1710.5	3267.4	4516.1	1645.3	4552.9	2488.8		2677.7
Count of PLR	***10***	***7***	***17***	***13***	***3***	***16***	***6***	***1***	***7***
Average of SII	75.0	76.4	75.5	123.6	100.1	119.2	104.7	136.9	109.3
StdDev of SII	27.4	14.5	23.3	59.5	38.4	55.9	43.2		41.3
Count of SII	***10***	***7.0***	***17***	***13***	***3***	***16***	***6***	***1***	***7***

RBC red blood cells, **Ret** reticulocytes, **WBC** white blood cells, **Neu** neutriophils, **Lym** lymphocytes, **PLTs** platelets, **NLR** neutrophil-to-lymphocyte ratio. **PLR** platelet-to-lymphocyte ratio, **SII** systemic immune-inflammation index ((PLTs x Neu)/Lym).
